# Microbial Indoles: Key Regulators of Organ Growth and Metabolic Function

**DOI:** 10.3390/microorganisms12040719

**Published:** 2024-04-02

**Authors:** Peter Yuli Xing, Ruchi Agrawal, Anusha Jayaraman, Katherine Ann Martin, George Wei Zhang, Ee Ling Ngu, Llanto Elma Faylon, Staffan Kjelleberg, Scott A. Rice, Yulan Wang, Adesola T. Bello, Elaine Holmes, Jeremy K. Nicholson, Luke Whiley, Sven Pettersson

**Affiliations:** 1 Singapore Centre for Environmental Life Sciences Engineering, Singapore 637551, Singapore; 2 Interdisciplinary Graduate School, Nanyang Technological University, Singapore 637335, Singapore; 3 Lee Kong Chian School of Medicine, Nanyang Technological University, Singapore 308232, Singapore; 4 ASEAN Microbiome Nutrition Centre, National Neuroscience Institute, Singapore 308433, Singapore; 5 Faculty of Medical Sciences, Sunway University, Subang Jaya 47500, Selangor, Malaysia; 6 School of Biological Sciences, Nanyang Technological University, Singapore 637551, Singapore; 7 Singapore Phenome Centre, Singapore 636921, Singapore; 8 Department of Metabolism, Digestion and Reproduction, Imperial College London, London SW7 2AZ, UK; 9 UK Dementia Research Institute, Imperial College London, London W1T 7NF, UK; 10 Australian National Phenome Centre, Health Futures Institute, Murdoch University, Perth, WA 6150, Australia; 11 Institute of Global Health Innovation, Imperial College London, London SW7 2NA, UK; 12 Perron Institute, Nedlands, WA 6009, Australia; 13 Karolinska Institutet, 171 77 Solna, Sweden; 14 Department of Microbiology and Immunology, National University Singapore, Singapore 117545, Singapore

**Keywords:** germ-free, gut microbiota, host metabolism, indoles, organ function decline, oxidative stress

## Abstract

Gut microbes supporting body growth are known but the mechanisms are less well documented. Using the microbial tryptophan metabolite indole, known to regulate prokaryotic cell division and metabolic stress conditions, we mono-colonized germ-free (GF) mice with indole-producing wild-type *Escherichia coli* (*E. coli*) or tryptophanase-encoding tnaA knockout mutant indole-non-producing *E. coli*. Indole mutant *E. coli* mice showed multiorgan growth retardation and lower levels of glycogen, cholesterol, triglycerides, and glucose, resulting in an energy deficiency *despite* increased food intake. Detailed analysis revealed a malfunctioning intestine, enlarged cecum, and reduced numbers of enterochromaffin cells, correlating with a metabolic phenotype consisting of impaired gut motility, diminished digestion, and lower energy harvest. Furthermore, indole mutant mice displayed reduction in serum levels of tricarboxylic acid (TCA) cycle intermediates and lipids. In stark contrast, a massive increase in serum melatonin was observed—frequently associated with accelerated oxidative stress and mitochondrial dysfunction. This observational report discloses functional roles of microbe-derived indoles regulating multiple organ functions and extends our previous report of indole-linked regulation of adult neurogenesis. Since indoles decline by age, these results imply a correlation with age-linked organ decline and levels of indoles. Interestingly, increased levels of indole-3-acetic acid, a known indole metabolite, have been shown to correlate with younger biological age, further supporting a link between biological age and levels of microbe-derived indole metabolites. The results presented in this resource paper will be useful for the future design of food intervention studies to reduce accelerated age-linked organ decline.

## 1. Introduction

Bidirectional host–gut microbiome interactions occur via two-way chemical communication through diverse molecules, including host-derived antimicrobial peptides and microbe-generated short-chain fatty acids (SCFAs) [1]. Indole(s) are tryptophan metabolites derived from gut microbe metabolism of dietary tryptophan through tryptophanase (encoded by the *tnaA* gene), exemplified by *Bacteroides thethaiotamicron* and *Escherichia coli* (*E. coli*) [2], that have direct physiological consequences on the host such as inhibiting inflammatory processes or enhancing gut barrier integrity through activation of the aryl hydrocarbon receptor (AhR) signaling pathways [3]. Germ-free (GF) mice have very low levels of indoles due to a lack of gut microbiota [4]. In contrast, indole concentrations in healthy mice and humans have been reported to be about 80 nmol/g and 145 nmol/g of fecal samples, respectively [5,6]. Indoles have been shown to extend the health span of flies, worms, and rodents based on their enhanced ability to maintain higher composite health metrics (e.g., motility, tolerance to stressors), reproductive ability, and more youthful genetic expression profiles [7]. Indole and indole-3-acetate (I3A) are important regulators of metabolic homeostasis; indole regulates glucose metabolism through colonic enteroendocrine L cells in vitro [8], whereas I3A inhibits hepatic gluconeogenesis and attenuates cytokine-mediated lipogenesis [9]. Interestingly, the systemic indole concentration inversely correlates with body mass index and obesity in human subjects and mice [10,11]. These observations are consistent with a model whereby gut microbe indole production is part of mammalian machinery regulating organ homeostasis, relevant to the aging body. 

Reduced indole levels in adult humans with metabolic syndrome/obesity and rats fed on a high-fat diet suggest a correlation between gut microbial indole production and metabolic homeostasis [12,13]. We used a reductionist approach and introduced a mutation in *E. coli* by removing the tryptophanase-encoding gene *tnaA* (*ΔtnaA*), thereby eliminating the conversion of tryptophan into indoles by these bacteria. GF mice were colonized with wild-type (indole-producing) or *ΔtnaA* (indole-non-producing) *E. coli* (hereafter referred to as WT and MT mice, respectively). This approach left the eukaryotic genetic phenotype intact, enabling us to monitor the interplay between organ function and gut microbes, with and without the ability to produce indoles. 

## 2. Materials and Methods

### 2.1. Animal Experiments

All animal experiments were carried out with the approval of the Institutional Animal Care and Use Committee (IACUC) of the Nanyang Technological University (NTU), Singapore (reference numbers: AUP-E0016 and AUP-A19104) and SingHealth, Singapore (reference number: 2016/SHS/1263), and in accordance with the guidelines of the Responsible Care and Use of Laboratory Animals (RCULA) set out by the National Advisory Committee for Laboratory Animal Research (NACLAR) in Singapore. All mice were housed in designated rooms at the NTU Animal Research Facility (NTU-ARF) and the SingHealth Experimental Medicine Centre (SEMC), with appropriate temperature (20–24 °C) and humidity (40–60%), a 12 h light–dark cycle, and ad libitum food and water intake, unless otherwise specified.

Germ-free (GF) C57BL/6J mice aged 6–8 weeks were colonized with wild-type (indole-producing) or *tnaA* mutant (indole-non-producing) *E coli*. Based on the weight of the mice, 100–250 μL of the bacterial suspension was administered to each mouse by oral gavage. After gavage, a maximum of 5 mice were housed in each GF bubble tent. Forty-eight hours, 1 week, and 2 weeks after gavage, mouse body weights were recorded, and stool samples were collected. Suspensions of stool samples were streaked on bacterial culture agar plates to examine colonization and contamination. In addition, Kovac indole tests on WT and MT *E. coli* samples were conducted, where the absence of the pink-color indole ring in the MT sample suggested a lack of indole production. Once colonization in the mice was confirmed, male and female mice were paired at a ratio of 1:2 at 8 weeks of age for breeding. Pups were weaned at 3 weeks of age and monitored for growth characteristics. This study was conducted using only male mice. At the end points (~14 weeks), the gut, liver, muscle, adipose tissue, brain, and blood were harvested for further processing and analysis. 

### 2.2. Bacterial Culture

Wild-type and *tnaA* (encoding tryptophanase) mutant *E coli* strain K12 were kind gifts from Dr. Thomas K. Wood (Pennsylvania State University, Pennsylvania, USA) [14,15]. Bacteria were cultured in LB medium until the log phase. Subsequently, the log-phase bacteria were pelleted and resuspended in fresh LB medium to an optical density of 1 at 600 nm (OD600 1 = ~109 cfu/mL) for administration to GF mice.

### 2.3. Metabolic Chamber Assessment

PhenoMaster (TSE Systems, Bad Homburg, Germany) equipment was used for metabolic chamber assessment. Briefly, the mice (~13 weeks old) were transferred into the calorimetric chamber for immediate recording for 6 days, with the first 2.5 days serving as the habituation period and the following 3 days for the analysis. Mice had ad libitum access to food and water and were under a 12 h light/dark cycle, a constant temperature of 22 °C, and humidity of 67%. Oxygen consumption, carbon dioxide production, food and water consumption, and body weight were recorded.

### 2.4. LC-MS Targeted Metabolomic Analysis

All blood serum samples (10 WT and 10 MT) were prepared and analyzed using a previously established method [16]. Briefly, 30 µL of serum was spiked with stable isotope-labeled internal standards prior to protein precipitation and removal, which was performed using a two-step protocol consisting of the addition of methanol followed by bypass-through solid-phase extraction. Samples were then taken to dryness under nitrogen and resuspended in 10 mM ammonium formate with 0.5% formic acid. Five microliters were injected into a Waters Acquity UHPLC system coupled to a Waters TQ-S tandem mass spectrometer (UHPLC-MS/MS;Waters, Wilmslow, UK). Chromatographic separation was performed on a reversed-phase Waters Acquity HSS T3 C18 column (Waters, Wilmslow, UK; 150 mm × 2.1 mm × 1.8 µm) using an optimized 7 min solvent gradient. 

The spectral data were pre-processed using TargetLynx (Waters, Wilmslow, UK) to convert the LC-MS raw data into peak area ratios with the peak areas of internal standards. Calibration standards of known concentrations were used to generate a linear calibration plot. Quality control was performed using samples prepared with known concentrations of each metabolite and was used to assess the accuracy and precision of the analysis throughout the data acquisition.

### 2.5. NMR Sample Preparation, Data Acquisition, and Data Analysis

NMR spectroscopy was performed according to an established protocol [17]. For plasma preparation, 100 μL of plasma was mixed with 50 μL of serum buffer (75 mM NaH2HPO4, pH = 7.4, 100% D2O, 2 mM sodium azide, and 0.08% TSP sodium salt), vortexed, and 150 μL samples were transferred into a 3 mm outer-diameter NMR tube. NMR spectra were collected on a Bruker AVANCE III NMR spectrometer operating at 600.13 MHz with a BBI probe (Bruker Biospin, Ettlingen, Germany) at 310 K. For each sample, a Carr–Purcell–Meiboom–Gill spectrum was obtained with the sequence ((RD-90°-(τ-180°-τ) n-acquisition); τ = 300 μs, n = 128). The relaxation delay (RD) was 4 s, a 90° pulse was set at 7.6 μs (−11.03 db), and 32 free induction decays (FIDs) were used with 72 K data points and 20 ppm spectral width. For all spectral acquisition, the free induction decay NMR signals were multiplied by an exponential factor to give a line broadening of 0.3 Hz and were Fourier-transformed to obtain the usual frequency spectrum (TOPSPIN 3.5 software, Bruker Biospin, Rheinstetten, Germany). The spectra were automatically phased, baseline-corrected, and calibrated using the glucose signal at δ 5.23 ppm. 1H NMR spectral peaks were analyzed using Student’s *t*-test for univariate analysis with *p* < 0.05, which was regarded as significant.

### 2.6. Histological Analysis

The intestinal, adipose, and liver tissues were fixed in 4% formaldehyde, embedded in paraffin, and sectioned for histological analysis. The intestines were Swiss-rolled before paraffin embedding was performed. Similar portions of the jejuna were used for the analysis in all cases. Tissue sections were cut at 4 μm thickness with a microtome (Leica HistoCore MULTICUT, Leica Biosystems, Milton Keyens, UK) and stained with hematoxylin and eosin (H&E). Brightfield images of the sections were obtained using a Zeiss Axio Scan.Z1 slide scanner at 20× magnification, and subsequently analyzed with Zeiss ZEN Desktop microscope software (v3.0) (Carl Zeiss Microscopy GmbH, Oberkochen, Germany) and ImageJ software (v1.51s) (Image J, Bethesda, MD, USA) for quantification. For determining the size distribution and cell number of adipocytes, Adiposoft and Cell Counter plugins in ImageJ were used.

For goblet cell quantification, paraffin-embedded intestinal sections were deparaffinized, rehydrated, and stained with periodic acid–Schiff (PAS) reagent. Hematoxylin was used to counterstain nuclei. Sections were imaged using a Zeiss Axio Scan.Z1 slide scanner with a 20× objective and analyzed using Zeiss ZEN software (v3.0). The PAS-stained goblet cells were counted per villus.

Liver tissues were fixed with 4% paraformaldehyde, equilibrated in 20% and 30% sucrose solutions, and sectioned at 8 μm thickness using a Leica CM3050-S cryostat ( Leica Biosystems, Milton Keyens, UK) The tissue sections were rinsed with 60% isopropanol, stained with freshly prepared Oil Red O (Sigma-Aldrich O0625-25G, St. Louis, MO, USA) solution, and lightly counter-stained with hematoxylin. Stained sections were imaged with a Zeiss Axio Scan.Z1 slide scanner with a 20× objective and analyzed using Zeiss ZEN software (V3.0).

### 2.7. Immunohistochemistry

Paraffin-embedded intestinal tissue sections were deparaffinized, rehydrated, and heat-treated in antigen-retrieval buffer (sodium citrate buffer or Tris-EDTA-Tween 20 buffer). For KI67 labeling, the sections were incubated with either rabbit polyclonal antibody (diluted 1:800, Abcam ab15580, Abcam Limited, Cambridge, UK) or rabbit polyclonal antibody (diluted 1:800, Abcam ab45179). The sections were then incubated with ImmPRESS reagent (Vector, HRP-conjugated secondary antibodies, and DAB substrate (Vector SK-4100, Vector Laboratories, Newark, CA, USA), and counterstained with hematoxylin. Sections were imaged using a Zeiss Axio Scan.Z1 slide scanner at 20× magnification and analyzed using Zeiss ZEN software. 

### 2.8. Quantitative Real-Time PCR

Total RNA was extracted from tissues using the Qiagen RNeasy Mini Kit (Qiagen 74106, Qiagen Sciences, Germantown. MD, USA) and used for complementary DNA synthesis with iScript Reverse Transcription Supermix (Bio-Rad 1708840, Life Science, Hercules, CA, USA), according to the manufacturer’s protocols. mRNA expression was quantified with Applied Biosystems StepOnePlus Real-Time PCR Systems, using Fast SYBR Green Master Mix (Applied Biosystems 4385612, San francisco, CA, USA) or Roche Lightcycler 96, using the iTaq SYBR Green one-step kit (Bio-Rad) and DNA oligo primers for targeted genes. At least six biological replicates were used for the analysis in triplicate. Mouse *Hprt1* was used as the reference gene for the normalization of expression. Relative quantification of mRNA levels from various treated samples was determined by the comparative Ct method [18]. The DNA oligonucleotide primer sequences are listed in Table S5.

### 2.9. Western Blotting

The tissues were homogenized in RIPA lysis buffer (Abcam ab156034, supplemented with phosphatase inhibitor cocktail, Roche 04906845001) for protein extraction. Bradford protein assay was used to determine protein concentration with standard curves established using BSA solution. A fixed amount of the protein extract was subjected to electrophoresis and transferred to nitrocellulose membranes. The membranes were incubated for 1 h in 5% BSA at room temperature and incubated overnight at 4 °C with the appropriate primary antibodies. Target proteins were detected by various primary antibodies (for phosphor-CREB rabbit mAb, Cell Signaling Technology #9198 (Danvers, MA, USA); for CREB rabbit mAb, Cell Signaling Technology #4820 (Danvers, MA, USA); for normalizing control β-Actin mouse mAb, Santa Cruz sc-47778 (Dallas, TX, USA)), with HRP-conjugated secondary antibodies (Goat Anti-Rabbit Immunoglobulins/HRP, Dako P0448 (Santa Clara, CA, USA); Horse Anti-Mouse IgG, HRP-linked, Cell Signaling Technology #7076 (Danvers, MA, USA)). The BCIP/NBT substrate was used for detection. The results were quantified using a Bio-Rad ChemiDoc Imaging System (Life Science, Hercules, CA, USA) and analyzed using ImageJ software.

## 3. Results

### 3.1. Indole Mutant Mice Display Reduced Body Weight and Weight Reduction across Multiple Organs

**Figure 1:**
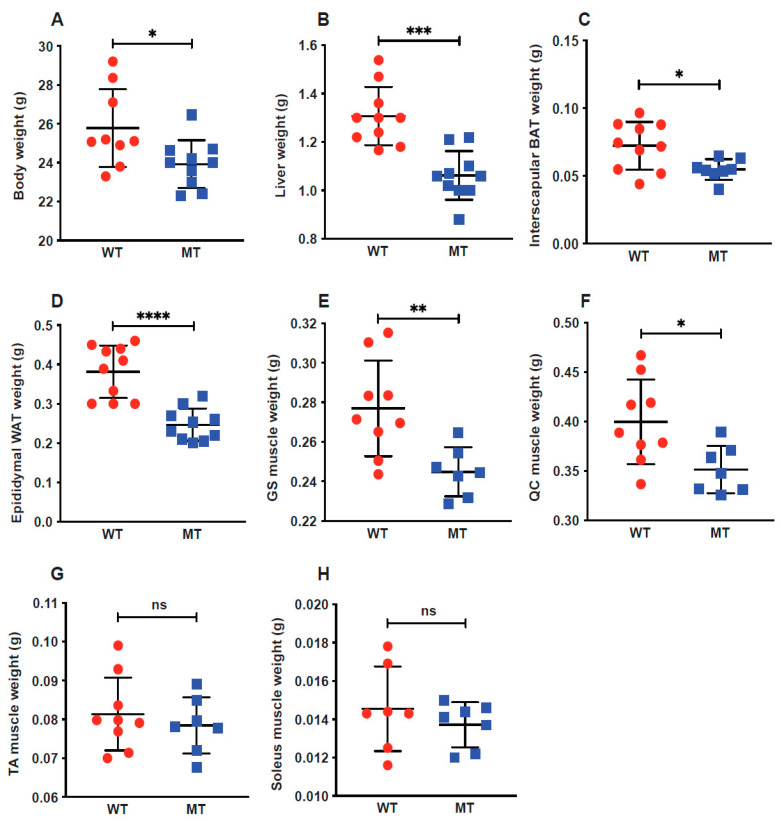
Germ-free (GF) mice colonized with indole-non-producing *E. coli* display lower body weights and lighter metabolic organs. Six–eight-week-old GF mice were colonized with wild-type (indole-producing, WT) and tnaA mutant (indole-non-producing, MT) *E. coli* by oral gavage. (**A**) Body weight of WT (n = 9) and MT (n = 10) mice at harvest. (**B**–**H**) Weights of WT and MT mouse livers (**C**), interscapular brown adipose tissue (BAT) (**D**), epididymal white adipose tissue (WAT) (**E**), hindlimb gastrocnemius muscle (GS) (**F**), and quadriceps (QC) (**G**), tibialis anterior (TA) (**G**) and soleus (**H**) (n = 7–10 per group). Data graphs show the mean ± SEM error bars; *p* values were calculated using Student’s *t*-test. Statistical significance between the indicated groups is presented as * *p* < 0.05, ** *p* < 0.01, *** *p* < 0.001, and **** *p* < 0.0001.

MT mice showed a reduction in body weight at the adult stage (measured at ~14 weeks) compared to WT mice (Figure 1A). Significant reduction in gross weight was also observed in indole mutant mice across the main metabolic organs: the liver, interscapular brown adipose tissue (BAT), epididymal white adipose tissue (WAT), and hindlimb gastrocnemius (GS) and quadriceps (QC) muscles (Figure 1B–F). The weights of two other hindlimb muscles, the tibialis anterior (TA, fast twitch) and soleus (slow twitch), also tended to be reduced, but not significantly (Figure 1G,H). These changes were not accompanied by any evidence of atrophy, as observed by histological analyses (Figure S1). Notably, these changes in different organs were observed in the indole mutant mice, which were genetically intact germ-free mice.

### 3.2. Indole Mutant Mice Display Alterations in Serum Metabolite Profiles

**Figure 2:**
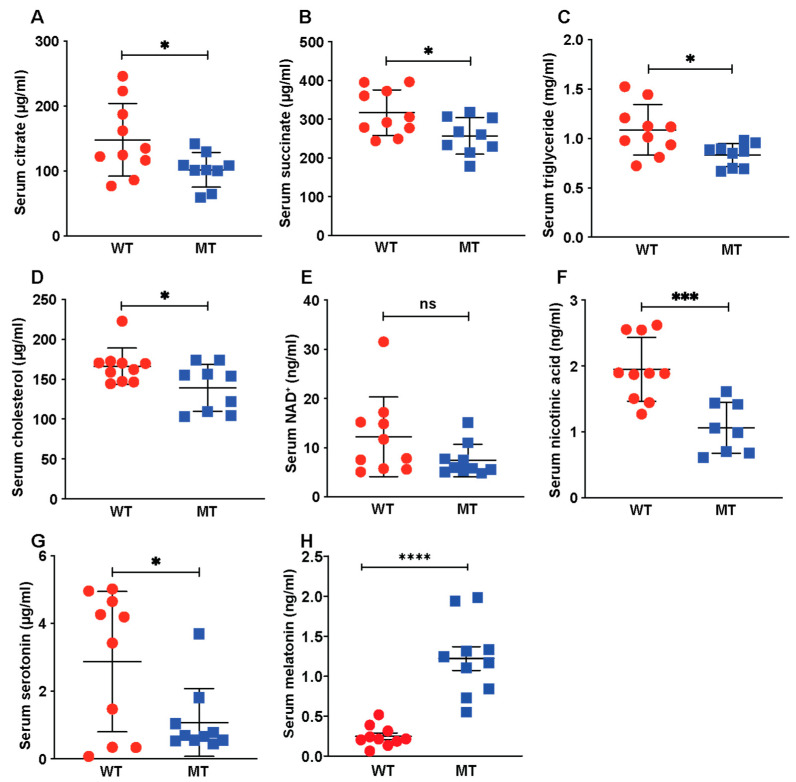
Loss of gut microbial indole production reduced concentrations of tricarboxylic acid (TCA) cycle intermediates, lipids, and serotonin in mouse serum. (**A**,**B**) Serum levels of TCA cycle intermediates, citrate and succinate, in WT (n = 10) and MT (n = 9) mice. (**C**) Serum nicotinamide adenine dinucleotide (NAD^+^) levels in WT and MT mice (n = 10 per group). (**D**) Serum levels of nicotinic acid (also known as niacin or vitamin B3) in the WT (n = 10) and MT (n = 8) mice. (**E**,**F**) Serum levels of triglycerides and cholesterol in WT (n = 10) and MT (n = 9) mice. (**G**) Serum serotonin levels in WT and MT mice (n = 10 per group). (**H**) Serum melatonin levels in WT and MT mice (n = 10 per group). (**A**–**H**), Data graphs show means ± S.D; *p* values were calculated using Student’s *t*-test. Statistical significance between the indicated groups is presented as * *p* < 0.05, *** *p* < 0.001 and **** *p* < 0.0001; ns denotes not significant and *p* ≥ 0.05.

Next, we performed targeted metabolite profiling using a combination of liquid chromatography–mass spectrometry (LC-MS) and nuclear magnetic resonance (NMR) spectroscopic methods on a select set of metabolites involved in host metabolism as well as host and gut microbial tryptophan metabolism (Table 1). Compared with WT mice, MT mice had reduced levels of serum citrate and succinate (Figure 2A,B), two metabolic intermediates in the tricarboxylic acid (TCA) cycle. The TCA cycle converts acetyl-CoA from glycolysis to nicotinamide adenine dinucleotide (NADH) for oxidative phosphorylation and energy harvesting. MT mice also had lower serum triglyceride and cholesterol levels (Figure 2C,D). Taken together, these data imply an impairment in mitochondrial metabolic activities, as both the TCA cycle and β-oxidation of fatty acids occur in the mitochondria. Interestingly, NAD+, the cofactor carrying electrons from glycolysis and the TCA cycle for ATP generation by oxidative phosphorylation in mitochondria, was present at lower levels in the serum of MT mice, although not significantly different (Figure 2E). However, there was a significant reduction in the serum levels of nicotinic acid in the MT mice (Figure 2F), which may have contributed to the reduced serum NAD+ levels. We found lower serum levels of the neurotransmitter serotonin (Figure 2G) and dopamine and dopamine metabolites (Table 1) in the MT, coupled with higher levels of the neuronal hormone melatonin (Figure 2H).

### 3.3. Indole Mutant Mice Display Alterations in Host Liver Metabolic Functions, Mitochondrial Functions, and Lipid Synthesis

**Figure 3:**
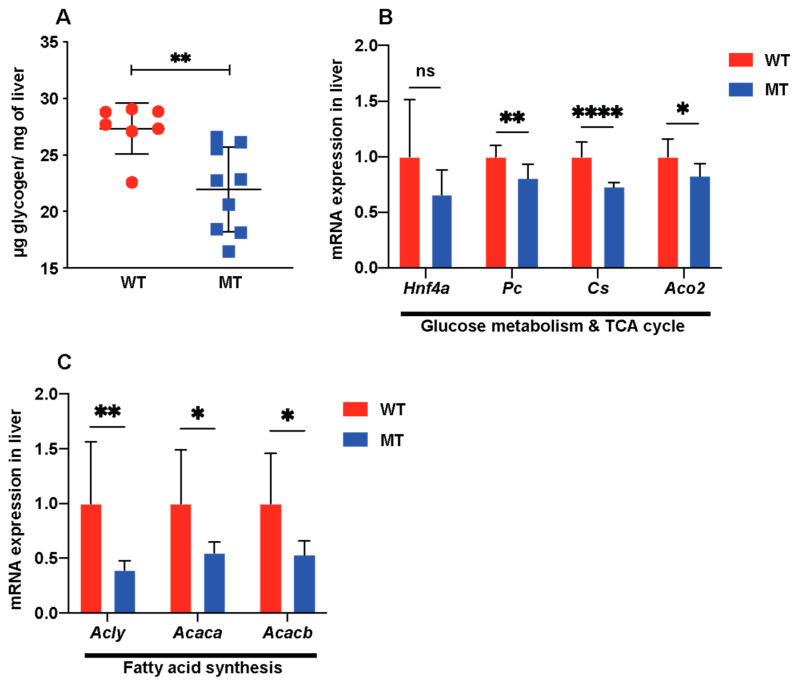
Indole-non-producing *E. coli*-colonized mice show signs of reduced glucose metabolism and lipogenesis in livers. (**A**) Liver glycogen levels (μg glycogen per mg tissue) (WT, n = 7; MT, n = 9). (**B**) mRNA expression of glucose metabolism regulator Hnf4a, TCA cycle enzymes Pc (pyruvate carboxylase), Cs (citrate synthase), and Aco2 (aconitase 2) in liver tissues of WT and MT mice (n = 8 per group). (**C**) mRNA expression of ATP citrate lyase (Acly, converting citrate to acetyl-CoA) and acetyl-CoA carboxylase (Acaca and Acacb, converting acetyl-CoA to malonyl-CoA, the rate-limiting step in fatty acid synthesis) in the livers of WT and MT mice (n = 7–8 per group). The mRNA levels were normalized to those of the control gene, Hprt1. (**A**–**C**), Data graphs show means ± S.D; *p* values were calculated using Student’s *t*-test. Statistical significance between the indicated groups is presented as * *p* < 0.05, ** *p* < 0.01, and **** *p* < 0.0001; ns denotes not significant and *p* ≥ 0.05.

As the serum levels of key metabolites were reduced in MT mice, to gain deeper insight into the effect of microbial indole production on metabolic processes, we processed and analyzed the main metabolic organs: liver, WAT, and muscles. We did not observe a significant difference in liver histology between the two groups (Figure S1), suggesting lack of any underlying organ atrophy in the MT mice. The livers of MT mice contained lower levels of glycogen than those of WT mice (Figure 3A). Hepatocyte nuclear factor-4α (Hnf4a), a regulator of genes involved in glucose transport and glycolysis, was expressed at slightly lower levels in the livers of MT mice than in those of WT mice (Figure 3B). The expression levels of enzymes involved in the TCA cycle (Pc, Cs, Aco2) and lipogenesis (Acly, Acaca, Acacb) were significantly reduced in the MT mouse liver (Figure 3B,C), which was consistent with the reduced serum levels of citrate and succinate (Figure 2A,B).

**Figure 4:**
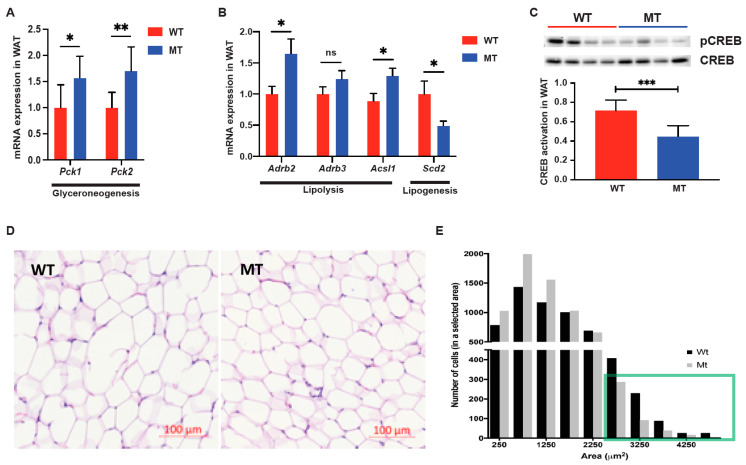
White adipose tissue (WAT) of indole-non-producing *E. coli*-colonized mice showed elevated glyceroneogenesis and lipolysis. (**A**) mRNA expression of Pck1 and Pck2 (phosphoenolpyruvate carboxykinase 1/2, cytosolic/mitochondrial, respectively) in the epididymal WAT of WT and MT mice (n = 7–8 per group). Phosphoenolpyruvate carboxykinases catalyze a rate-limiting step in glyceroneogenesis in WAT, which generates glycerol 3-phosphate from precursors other than glucose during fasting. (**B**) mRNA expression of lipolysis regulators Adrb2/3 (β_2/3_ adrenoreceptor), long-chain fatty acid coenzyme A ligase Acsl1, and fatty acid synthesis enzyme stearoyl-CoA desaturase (Scd2) in epididymal WAT of WT and MT mice (n = 6–8 per group). The mRNA levels were normalized to those of the control gene, Hprt1. (**C**) Western blotting and quantification of activation of cyclic AMP-response element-binding protein (CREB), a lipogenesis regulator, in epididymal WAT of WT and MT mice (n = 6–8 per group). Phosphorylated CREB (pCREB), a measure of CREB activation, was normalized to the total CREB. (**D**) Representative images of epididymal white adipose tissue (WAT) histological analyses by hematoxylin and eosin (H&E). Scale bars = 100 μm. (**E**) Histogram of adipocyte areas. The green box shows that there is a lower number of bigger adipocytes in the MT mice than in the WT mice. (**A**–**C**), Data graphs show means ± S.D; *p* values were calculated using Student’s *t*-test. Statistical significance between the indicated groups is presented as * *p* < 0.05, ** *p* < 0.01 and *** *p* < 0.001; ns denotes not significant and *p* ≥ 0.05.

Interestingly, in the epididymal WAT of MT mice, the mRNA levels of the glyceroneogenesis rate-limiting enzyme Pck1 and another glyceroneogenesis enzyme Pck2 were upregulated (Figure 4A). Glyceroneogenesis, which generates glycerol 3-phosphate from precursors other than glucose, such as pyruvate, typically occurs during fasting or starvation in WAT [19]. The production of glycerol 3-phosphate enables the re-esterification of free fatty acids in adipose tissue, which is a concomitant process with active lipolysis [20]. Consistently, the mRNA levels of regulators of lipolysis Adrb2/3, as well as long-chain fatty acid coenzyme A ligase Acsl1, were significantly upregulated in MT mice (Figure 4B). Lipogenesis enzyme mRNA expression was also downregulated in the WAT (Scd2, Figure 4B) of MT mice. Coupled with the upregulation of lipolysis genes, decreased activation of cyclic AMP-response element-binding protein (CREB), a primary regulator of lipogenesis, was observed in the WAT of MT mice (Figure 4C). Corresponding to the reduced weight of WAT in MT mice (Figure 1E), the morphological analysis of adipocytes showed more cells with smaller areas and a reduced number of cells with larger areas, compared to WT samples (Figure 4D,E). These results suggest that MT mice have elevated lipolysis in WAT than WT mice, indicative of metabolic stress in the indole mutant mice.

**Figure 5:**
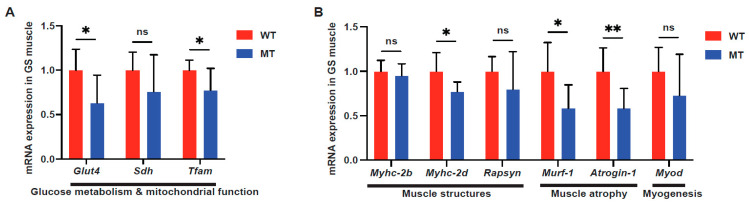
Indole-non-producing *E. coli*-colonized mice contained reduced glycogen levels in the gastrocnemius muscles. (**A**) mRNA expression of the glucose transporter Glut4, the TCA cycle, and the respiratory chain enzyme succinate dehydrogenase Sdh, and a key activator of mitochondrial transcription, Tfam (mitochondrial transcription factor A (Tfam), in hindlimb gastrocnemius (GS) muscle tissues of WT and MT mice (n = 7–8 per group). (**B**) mRNA expression of myosin heavy-chain II proteins Myhc-2b and Myhc-2d, neuromuscular junction-associated gene Rapsyn (43 kDa receptor-associated protein of the synapse), muscle atrophy E3 ubiquitin ligases Murf-1 (E3 ubiquitin-protein ligase TRIM63) and Atrogin-1 (F-box only protein 32), and myogenesis-related gene Myod (myoblast determination protein 1) in GS muscles of WT and MT mice; (n = 7–8 per group). mRNA levels were normalized to the control gene Hprt1. Data graphs show means ± S.D; *p* values were calculated with Student’s *t*-test. Statistical significance is presented as * *p* < 0.05 and ** *p* < 0.01 between indicated groups; ns denotes not significant with *p* ≥ 0.05.

Glucose metabolism and mitochondrial functional genes [for example, Glut4 (a glucose transporter), succinate dehydrogenase (SDH; a TCA cycle and respiratory chain enzyme), and Tfam (a key activator of mitochondrial transcription)], all showed reduced RNA expression in GS (Figure 5A). Corresponding to the reduced glucose metabolism in the GS muscles of MT mice, the mRNA expression of myosin heavy-chain II proteins Myhc-2b and Myhc-2d, the “fast” isoforms of myosin heavy-chain proteins (i.e., utilizing glycolysis), was downregulated, along with Rapsyn, a neuromuscular junction-associated gene (Figure 5B). Notably, no signs of muscle atrophy were observed. Interestingly, the muscle atrophy-related genes Murf-1 and Atrogin-1 (encoding E3 ubiquitin ligases), and myogenesis-related gene MyoD, were downregulated in the GS muscles of GS muscle from MT mice (Figure 5B). The full list of genes analyzed in liver tissue, WAT, and hindlimb muscles is presented in Tables S1–S3.

### 3.4. Indole Mutant (MT) Mice Show Increased Food Intake and Locomotion through Calorimetric Cage Analysis

The low body weight, reduced serum levels of TCA cycle intermediates and lipids, signs of oxidative stress (increased melatonin level), lowered glycogen deposition in liver and muscle, combined with elevated lipolysis and reduced lipogenesis gene expression in the liver and adipose tissues, implied that the indole mutant mice displayed a metabolic stress phenotype. Decreased intestinal function to digest and absorb nutrients or increased metabolic rate (spending more energy) could be the underlying cause. Therefore, we performed metabolic cage experiments to monitor food and water intake, oxygen consumption, carbon dioxide production, heat production, and locomotion at room temperature (22 °C). 

**Figure 6:**
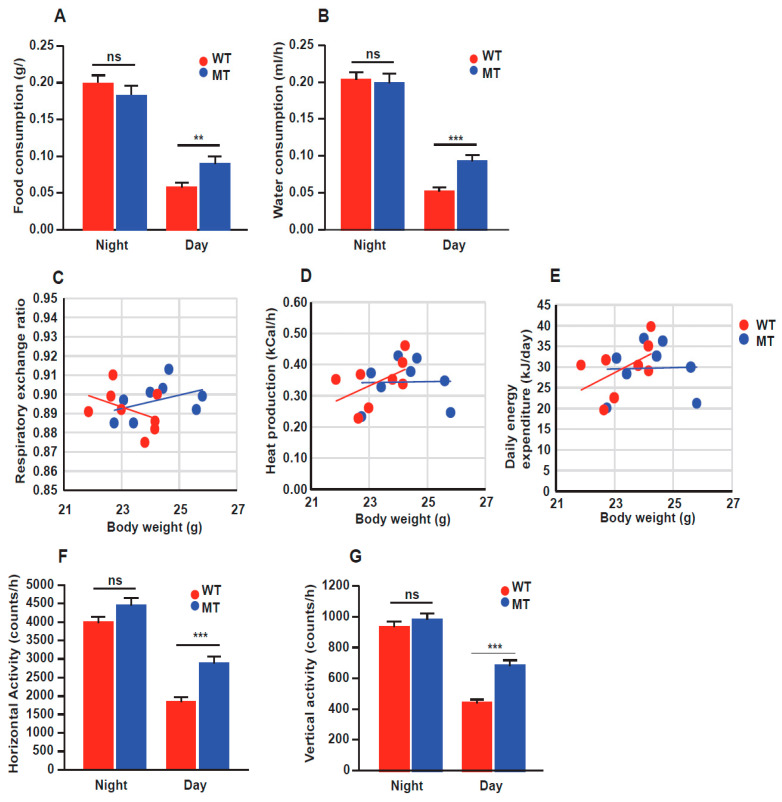
Mice with indole-non-producing *E. coli* showed increased food intake and locomotor activity through calorimetric cage analysis. (**A**,**B**) Food and water consumption at night- and daytime of WT and MT mice. (**C**–**E**) Linear regression curves for correlation of body weight with the respiratory exchange rates, heat production, and daily energy expenditure. Data are the means of results over three experimental days per animal. Each dot represents an animal. (**F**,**G**) Horizontal and vertical activities of WT and MT mice. Experiments were conducted at a constant temperature of 22 °C and humidity of 67%. A total of 3 days in the calorimetric cages were used for the data analysis; n = 8 per group. For (**A**,**B**,**F**,**G**), data graphs show means ± SEM error bars; *p* values were calculated with ANOVA (Welch’s robust *t*-test of equality of means). Statistical significance is presented as ** *p* < 0.01, and *** *p* < 0.001 between indicated groups; ns denotes not significant with *p* ≥ 0.05. For (**C**–**E**), data were analyzed by ANCOVA GLM statistical analysis comparing the two groups with the body weight and day/night variation as covariates.

MT mice exhibited significantly elevated food consumption during the daytime compared to WT mice, although they had similar food and water intake at night (Figure 6A,B). Interestingly, the respiratory exchange rate, heat production, and calculated daily energy expenditure did not differ between the two groups (Figure 6C–E). However, we noticed an increase in the locomotor activity in the MT mouse group (Figure 6F,G), although this difference was not correspondingly reflected in the daily energy expenditure.

### 3.5. Indole Mutant (MT) Mice Have Increased Intestinal Length, Enlarged Cecum, Increased Epithelial Cell Growth, and Reduced Serotonin-Producing Colonic Enterochromaffin Cells

**Figure 7:**
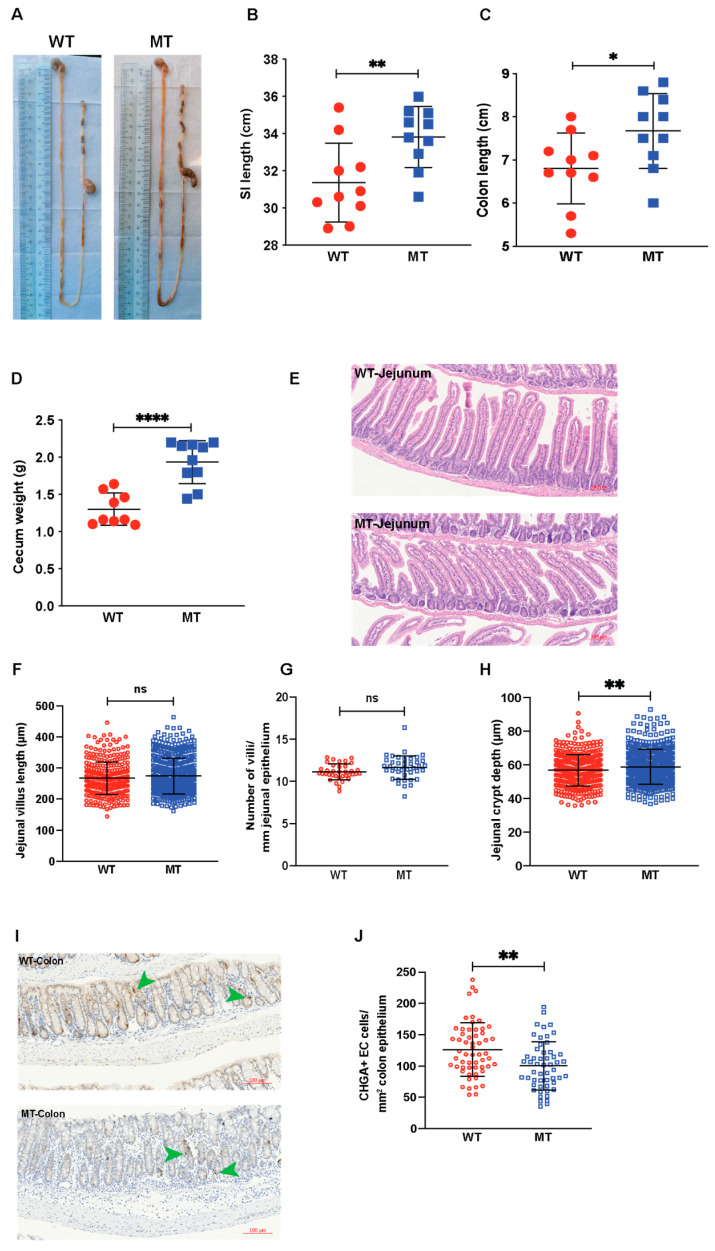
Mice with indole-non-producing *E. coli* showed enlarged cecum without drastic changes in general histology. (**A**) Representative images of the harvested gastrointestinal tracts of WT and MT mice. (**B**–**D**) Lengths of the small intestine (SI, (**B**)) and colon (**C**), and weight of ceca (**D**) of WT and MT mice (n = 9–10). (**E)** Representative images of hematoxylin and eosin (H&E) staining of the jejuna from WT and MT mice. Scale bar, 100 μm. (**F**,**H**) Quantification of villus length (**F**), villus number (**G**), and crypt depth (**H**) in the jejuna of WT and MT mice (n = 6 per group). (**I**) Representative images of colonic tissue sections stained with anti-chromogranin A (CHGA) antibody for enterochromaffin cells (brown, indicated by green arrowheads) of WT and MT mice. Nuclei were counterstained with hematoxylin (blue). Scale bar, 100 μm. (**J**) Quantification of CHGA^+^ enterochromaffin cells (number of cells per mm^2^ of colonic epithelium) in the colons of WT and MT mice (n = 6 per group). Data graphs show means ± S.D; *p* values were calculated using Student’s *t*-test. Statistical significance between the indicated groups is presented as * *p* < 0.05, ** *p* < 0.01, and **** *p* < 0.0001; ns denotes not significant and *p* ≥ 0.05.

As the results thus far show that MT mice were eating more and not burning more energy, but still had a reduced body weight, we next monitored the intestinal morphology including cecum area to investigate a possible digestion and/or absorption problem in the intestinal tract. Indeed, MT mice had a longer small intestine (SI) and colon (Figure 7A–C) and an enlarged cecum filled with semi-digested food (Figure 7D). Further histological analysis of the jejunal epithelium showed no difference in the villi length and number (Figure 7E–G), but significant changes were observed in the crypt depths (Figure 7H). Taken together, these findings suggest that the MT mice have problems accessing nutrients either by an impairment in digestion and absorption or a combination of both. The increase in SI length could be a compensatory mechanism in the MT mice to increase the surface area of epithelial cells to overcome the malabsorption problems and facilitate more absorption of nutrients. 

Serotonin in the gut is known to regulate colon motility [21], and having observed reduced serotonin levels in the serum of MT mice (Figure 2G), we quantified the enterochromaffin (chromogranin A (CHGA)-expressing, CHGA^+^) cells in the SI and colon by immunohistochemistry. We found a significant reduction in the number of CHGA^+^ cells in the colon, but not in the SI of MT mice (Figure 7I,J and Figure S2). This skewed morphological phenotype combined with reduced gut motility and digestive function has been observed in mice with reduced serotonin levels [22]. Gene expression analysis of the enzymes involved in serotonin synthesis and degradation in the SI showed similar results between the two groups (Table S4).

Intestinal barrier integrity and inflammatory status also play crucial roles in normal digestive and absorptive functions [23]. Therefore, we screened a panel of barrier integrity and inflammation markers in the SI epithelium. We found no differences between WT and MT mice in the gene expression of the tight junction proteins Occludin, Zo-1, and Claudin-1, or in the pro-inflammatory cytokines Tnfa, Il1b, and Il18 between the two groups (Figure S3). 

**Figure 8:**
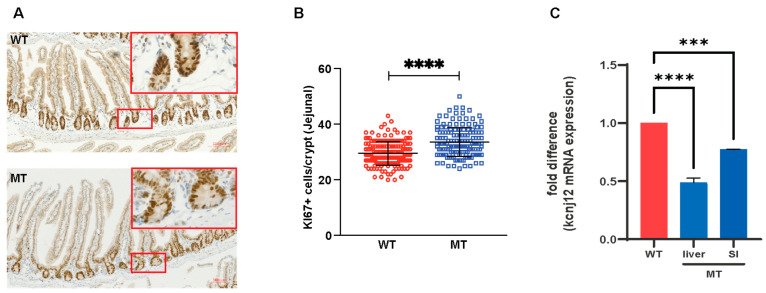
Mice colonized with indole-non-producing *E. coli* showed elevated SI epithelial growth. (**A**) Representative images of jejunal tissue sections stained with anti-KI67 antibody for proliferating cells (brown) in the jejuna of WT and MT mice with hematoxylin-counterstained nuclei (blue). Scale bar, 100 μm. (**B**) Quantification of proliferating KI67-expressing (KI67^+^) cells (number of cells per crypt) in the jejuna of WT and MT mice (n = 6 per group). (**C**) mRNA expression of Kcnj12 in the liver and small intestine (SI) of WT and MT mice (n = 8 per group). The mRNA levels were normalized to those of the control gene, Hprt1. Data graphs show means ± S.D; *p* values were calculated using Student’s *t*-test. Statistical significance between the indicated groups is presented as *** *p* < 0.001 and **** *p* < 0.0001.

As intestinal crypts contain proliferating stem cells and transit-amplifying progenitor cells [24], we performed immunohistochemical staining for the cellular proliferation marker KI67+, which confirmed a marked increase in the number of proliferating KI67+ cells in the SI of the MT mice (Figure 8A,B). As indoles are known to elicit cell cycle exit [25], this finding is consistent with the reduced levels observed in the MT mice. 

These findings were further addressed by evaluating the kcnj12, the inwardly rectifying K+ channel known to regulate gut motility (action potential waveform and excitability of neuronal tissues) and mitochondrial membrane potential and mitochondrial reactive oxygen species (ROS) production [26,27]. This gene is regulated by the indole–aryl hydrocarbon receptor (AhR) signaling pathway [28]. As can be seen, a considerably reduced kcnj12 expression in intestinal and liver tissue was observed in the MT mice (Figure 8C). These findings correlated with the reduced gut motility due to impairment in SI smooth muscle function and mitochondrial dysfunction in the liver, consistent with reduced levels of indoles in MT mice. 

Mucus-secreting goblet cells are a major group of secretory cells in the intestinal epithelium. Periodic acid–Schiff (PAS) staining of the intestines revealed a decrease in the number of goblet cells in the jejunal villi of MT mice (Figure S4A,B). However, no change in the mRNA expression of Muc2 was observed in the MT group (Figure S4C).

## 4. Discussion

We report that microbe-derived indoles regulate organ growth and functions. GF mice mono-colonized with indole-deficient microbes display growth retardation across multiple organs, considerable metabolic dysfunctions and reduced ability to access nutrients in the intestine, and an impairment in digestion of food. This is supported by reduced impairment in gut motility and enlargement of the cecum full of undigested food, thus resembling characteristic phenotypes displayed by GF and antibiotic-treated rodents, in which enlarged ceca are filled with undigested material [29]. Cecal enlargement induced by antibiotic treatment in mice is associated with reduced intestinal motility, impaired digestion, and longer gut transit time, which ultimately impacts energy harvesting and body weight [29,30]. Interestingly, aged mice often display reduced motility like that observed in our MT mice, and reduced indole levels associated with organ decline is observed in aging mice, implying a link between age-associated organ decline and levels of indoles [31]. Moreover, absorption problems of cholesterol in mice with increased crypt cell proliferation have been reported [32].

Previous studies have established the role of gut microbiota in the modulation of colonic enterochromaffin cell numbers and serotonin production and, thus, their role in gut motility and digestion [30,33]. The observations of increased intestinal length in indole mutant mice correlate with a possible compensatory mechanism to increase the absorption surface and capacity [34]. In addition, long-term exposure to antibiotics, which reduces the number of gut microbes and reduces serotonin levels, is reported to increase intestinal length, though with impairment in motility and function, similar to that observed in the indole mutant mice [30,34]. Furthermore, mono-colonization of GF mice exposed to *Clostridium ramosum* causes them to display increased levels of enterochromaffin cell differentiation and serotonin production, facilitating intestinal fatty acid absorption and modulating metabolic functions [35]. The indole mutant mice presented reduced serum serotonin and fewer colonic enterochromaffin cells, consistent with a dysfunctional intestine lacking the optimal ability to support serotonin production in the colon, thereby impairing intestinal functions relevant to digestion and the tuning of gut motility and digestion, and, therefore, host metabolic homeostasis. A recent report using mice with enteric neuron-specific deletion of AhR showed that the lack of AhR in these neurons resulted in significantly prolonged intestinal transit time (ITT) [26]. This observation from the Pachnis team is directly in line with our results in MT mice, suggesting reduced gut motility, as indoles are ligands for AhR activation [36]. Transcriptomics analysis of enteric neurons for downstream AhR targets that were also potential regulators of neuronal activity in these neurons identified kcnj12, a gene that encodes the inwardly rectifying K+ channel, subfamily J member 12 (Kir2.2) [26], which is known to regulate the excitability of cardiac muscles and neurons [37], and the gene we observed to be massively reduced in the intestine and liver of the MT mice (Figure 8C),. While speculative, we propose that this reduction in kcnj12 expression in the SI and livers of MT mice contributes, in part, to decrease the dysfunctional metabolic and intestinal phenotype observed in the MT mice. Notably, knockdown of *Kcnj12* has been shown to significantly increase ROS activation and trigger cell cycle arrest and mitochondria dysfunction [27]. 

In addition to the microbial metabolism of tryptophan that produces indole and indole derivatives, the host also metabolizes tryptophan through the kynurenine- and serotonin-producing pathways (Figure S5) [38]. The host kynurenine-producing pathway is upstream of the host de novo NAD+ synthesis, contributing to the NAD+ pool [38]. Apart from de novo NAD+ synthesis, the host may also produce NAD+ through salvage pathways, which utilize nicotinic acid (also known as niacin or vitamin B3) from dietary intake or gut microbial production. In addition to the reduction in serum levels of TCA cycle intermediates and lipids, altered concentrations of other metabolites were found in MT mice. Many of the biomarkers observed to change in the indole mutant mice were associated with increased ROS activity and/or impairment in mitochondrial function, including citrate synthase, the first enzyme in the TCA cycle, and a known quantitative indicator of intact mitochondria (Figure 2A) [39]. Melatonin is well known for its anti-oxidative stress effect by stimulating the production of endogenous antioxidant enzymes, superoxide dismutase (SOD), and glutathione peroxidase (GPx) [40,41], and the significant increase in serum melatonin in MT mice (Figure 2H and Table 1) further suggests elevated levels of ROS activity in indole mutant mice and could be a compensatory mechanism for the possible reduction in GPx production caused by mitochondrial pyruvate carrier (MPC) inhibition [42]. Gut microbes are known to regulate the transcription of tryptophan hydroxylase (TPH1) to catalyze a cascade of tryptophan metabolites formation, including melatonin and serotonin [43]. Moreover, melatonin has been reported to colocalize in the mitochondria—the major production site and target of ROS production [41]. Indoles are known to protect against oxidative stress [44]. Because ROS production increases with age, maintaining indole concentrations as one gets older may be one beneficial way to handle unwanted age-associated ROS levels [45]. Additionally, melatonin has been shown to delay the onset of puberty [46,47], which has been shown to result in lower skeletal muscle mass [48]. “Lipid cycling”, a combined process of lipolysis and glyceroneogenesis, was elevated in the MT mice. While further studies are required, the impairment in the MPC that led to reduced pyruvate being transported into the mitochondria may be one of several mechanisms to explain this finding [42]. An increase in the ATP demand then promotes the mitochondrial oxidative phosphorylation (OXPHOS) that is fueled by fatty acid (FA) oxidation [42]. Although further investigation is required, oxidative stress may play a role in the stimulation of lipolysis and lipid cycling by MPC inhibition, as mentioned above [42]. In this context, the downregulation of pyruvate carboxylase expression could also be explained by the MPC inhibition, but the reduction in citrate despite the availability of FA acetyl-CoA may be due to the downregulation of citrate synthase (CS) instead. Among the downregulated intermediates and enzymes involved in the TCA cycle and OXPHOS, citrate, succinate, and SDH are particularly highly relevant to oxidative stress. Despite being TCA intermediates, succinate (pro-oxidant) and citrate (antioxidant) have opposite roles in oxidative stress, whereas SDH oxidizes succinate to fumarate in the TCA cycle and acts as the electron transport chain (ETC) complex II, which is one of the major sites of ROS production [49].

Interestingly, metabolic alterations (including reduced levels of glycogen and increased expression of genes that promote mitochondrial OXPHOS) in the CS knock-down mouse model only occurred under fasting-mimic conditions [50]. The fact that our MT mice experienced fasting-mimic conditions (upregulated lipid cycling, downregulated lipogenesis and Glu4 expression) is consistent with the phenomena of mild insulin resistance [51]. This speculation is based on the increased lipolysis [52], as well as the ability of indole-3-acetic acid (IAA) to try to overcome insulin resistance [53]. Indole mutant mice displayed a massive reduction in plasma IAA levels (Table 1), thus hampering the ability of IAA to reduce lipogenesis and levels of triglyceride and cholesterol in mice fed with a high-fat diet (HFD) [53].

Another interesting observation is the increase in picolinic acid (PA) (Table 1), an endogenous metabolite of tryptophan known to have a beneficial effect by converting tryptophan to nicotinamide [54]. Our metabolomic analysis showed reduced levels of NAD+, though the reduction was not statistically significant (Table 1). However, increased levels of PA may be a compensatory mechanism in indole mutant mice.

## 5. Conclusions

We report that indole-producing microbes appear to be important for postnatal growth and metabolic functions. The previous observation that maintaining the indole levels in flies, worms, and mice is associated with a prolonged health span and that a reduction in indoles results in a shortening of the health span is consistent with our data, observed in the indole mutant mice [7]. The results presented here reflect a resource paper based on observations, and more detailed analyses are required to reveal the full scope of indole′s effects in regulating host metabolism and function. However, the reductionist approach of modifying the microbe rather than modifying host genetics provides an attractive alternative to gain an understanding of gut microbe–host interactions. Our model system—GF mice colonized with wild-type (indole-producing) or ΔtnaA (indole-non-producing) *E. coli*—let us specifically study the role of indole while leaving the eukaryotic genetic phenotype intact, enabling us to monitor the interplay between organ function and gut microbes, specifically with and without the ability to produce indoles. Based on the similarity of phenotypes between aged mice and MT mice, our findings from MT mice and about the potential underlying mechanisms could be extrapolated to understanding the aging process and provide insights into maintaining metabolic homeostasis. Gut microbe-derived tryptophan metabolites, indoles and I3A, are interesting representatives of the next-generation set of biomarkers to be considered as screening markers of accelerated organ decline as well as future nutritional supplementation to prolong the human health span and reduce organ decline.

## Figures and Tables

**Table 1:** Serum levels of selected metabolites by metabolomic studies; n = 8–10 per group; *p* values were calculated with Student’s *t*-test. Statistical significance is presented as * *p* < 0.05, ** *p* < 0.01, *** *p* < 0.001, and **** *p* < 0.0001 between indicated groups. ↑ represents increase in levels, ↓ represents decrease in levels, and - represents no change in levels in MT mice compared with WT mice.

Metabolic Pathways/ Physiological Roles	Molecules	Serum Levels in MT Mice Compared with WT Mice	Statistical Significance	*p* Values
Essential amino acid	Tryptophan	-	ns	0.7537
Host tryptophan metabolites—kynurenine-producing pathway	Kynurenine	↑	*	0.0405
	Kynurenic acid	-	ns	0.4083
	3OH-Kyn	-	ns	0.1140
	Xanthurenic acid	-	ns	0.7954
	3HAA	-	ns	0.5626
	Picolinic acid	↑	*	0.0398
	Quinolinic acid	-	ns	0.4603
	NAD^+^	-	ns	0.0970
Nicotinic acid metabolites	Nicotinic acid	↓	***	0.0007
	NMN	-	ns	0.1363
	NaR	-	ns	0.6148
Host tryptophan metabolites—serotonin-producing pathway	Serotonin	↓	*	0.0238
	5-HIAA	-	ns	0.0751
	Melatonin	↑	****	<0.0001
Microbial tryptophan metabolites	Tryptamine	-	ns	0.2705
	ILA	-	ns	0.1909
	IAA	↓	**	0.0055
TCA cycle intermediates	Succinate	↓	*	0.0262
	Citrate	↓	*	0.0381
Lipids	Triglycerides	↓	*	0.0139
	Cholesterol	↓	*	0.0348
Lipid digestion	Bile acids	↓	*	0.0426
Short-chain fatty acids	Formate	↑	**	0.0073
	Acetate	↓	*	0.0100
Amino acids	Glutamate	↓	*	0.0218
	Aspartate	↓	*	0.0229
	Phenylalanine	↓	*	0.0135
	Tyrosine	↓	*	0.0218
Dopamine/dopamine metabolites	Dopamine	↓	*	0.0138
	Dopamine-O-sulfate	↓	*	0.0219
	Dopamine-B-glucuronide	↓	***	0.0005
GTP catabolite	Neopterin	-	ns	0.0761
Urea cycle intermediate	Citrulline	-	ns	0.4277

## Data Availability

The original contributions presented in the study are included in the article/Supplementary Materials, and further inquiries can be directed to the corresponding author.

## Supplementary Materials

**The following supporting information can be downloaded at: https://www.mdpi.com/article/10.3390/microorganisms12040719/s1, Figure S1.** Representative images of liver histological analyses by (**A**) haematoxylin and eosin (H&E) and (**B**) oil red O staining. For **A**, scale bars = 100 μm; For **B**, scale bars = 200 μm; **Figure S2.** Jejunal serotonin-producing enterochromaffin cell numbers and serotonin metabolism in SI and liver in WT and MT mice. (A) Representative images of jejunal tissue sections stained with anti-chromogranin A (CHGA) antibody for enterochromaffin cells (in brown, indicated by green arrow heads), in jejuna of WT and MT mice, with haematoxylin-counterstained nuclei (in blue). Scale bar = 100 μm. (B) Quantification of CHGA+ enterochromaffin cells (number of cells per 10 villi) in jejuna of WT and MT mice (n = 6 per group). (C) mRNA expression of enzymes for serotonin synthesis Tph1 (tryptophan hydroxylase 1) and Ddc (5-hydroxytryptophan decarboxylase), serotonin transporter Slc6a4, and enzymes for serotonin metabolism Maoa and Aldh2, in the duodena of WT and MT mice (n = 8 per group). (D) mRNA expression of enzymes for serotonin metabolism Maoa and Aldh2 in the livers of WT and MT mice (n = 8 per group). mRNA levels were normalized to the control gene Hprt1. Data graphs show means ± S.D. *p* values were calculated with the student’s t test. ns denotes not significant with *p* ≥ 0.05 between indicated groups**; Figure S3.** qPCR analyses on (A) intestinal tight junction proteins, Occludin, Zo-1 (zonula occludens-1), and Claudin-1 (n = 8 per group); and (B) pro-inflammatory cytokines Tnfa (tumour necrosis factor alpha), Il1b (interleukin 1 beta), Il18 (interleukin 18), in the duodena of WT and MT mice (n = 7–8 per group). mRNA levels were normalized to the control gene Hprt1. Data graphs show means ± S.D. *p* values were calculated with the student’s t test. Statistical significance is presented as * *p* < 0.05 between indicated groups. ns denotes not significant with *p* ≥ 0.05; **Figure S4.** Indole-non-producing *E. coli*-colonized mice contained fewer mucus-producing goblet cells in the small intestines. (A) Representative images of jejunal tissue sections stained with periodic acid-Schiff (PAS) reagent for goblet cells (in deep purple, green arrow heads indicate representative cells), in jejuna of WT and MT mice, with haematoxylin-counterstained nuclei (in blue). Scale bar, 100 μm. (B) Quantification of goblet cells (number of PAS^+^ cells per villus) in jejuna of WT and MT mice (n = 6 per group). (C) mRNA expression of mucin 2 (*Muc2*) in the duodena of WT and MT mice (n = 8 per group). mRNA levels were normalized to the control gene *Hprt1*. Data graphs show means ± S.D. *p* values were calculated with the student’s *t* test. Statistical significance is presented as **** *p* < 0.0001 between indicated groups. ns denotes not significant with *p* ≥ 0.05; **Figure S5.** Illustration of tryptophan metabolisms in the mammalian host. Tryptophan can be metabolized in the host via the kynurenine pathway, through which kynurenine and NAD^+^, along with other metabolites, are produced. Alternatively, tryptophan can also be metabolized in the intestines by the enterochromaffin cells, or in the brain, through the serotonin pathway, leading to the production of serotonin. Serotonin can be further metabolized to 5-hydroxyindoleacetic acid (5-HIAA) or be utilized as a substrate for production of melatonin, a hormone regulating the sleep-wake cycle. NAD^+^, nicotinamide adenine dinucleotide (oxidized). Figure is adapted from Schwarcz & Stone, 2017^41^. Copyright © 2016 Elsevier Ltd. Adapted with permission; **Table S1:** mRNA expression levels of selected genes in liver samples from WT and MT mice. n = 7–8 per group. *p* values were calculated with the student’s *t* test. Statistical significance is presented as * *p* < 0.05, ** *p* < 0.01, *** *p* < 0.001, and **** *p* < 0.0001 between indicated groups. ns denotes no statistical significance with *p* ≥ 0.05**; Table S2.** mRNA expression levels of selected genes in epididymal white adipose tissue (WAT) samples from WT and MT mice. n = 6–8 per group. *p* values were calculated with the student’s *t* test. Statistical significance is presented as * *p* < 0.05, ** *p* < 0.01, *** *p* < 0.001, and **** *p* < 0.0001 between indicated groups. ns denotes no statistical significance with *p* ≥ 0.05; **Table S3.** mRNA expression levels of selected genes in hindlimb muscles gastrocnemius (GS) samples from WT and MT mice. n = 7–8 per group. *p* values were calculated with the student’s *t* test. Statistical significance is presented as * *p* < 0.05, ** *p* < 0.01, *** *p* < 0.001, and **** *p* < 0.0001 between indicated groups. ns denotes no statistical significance with *p* ≥ 0.05; **Table S4.** mRNA expression levels of selected genes in small intestine (SI) samples from WT and MT mice. n = 7–8 per group. *p* values were calculated with the student’s *t* test. Statistical significance is presented as * *p* < 0.05, ** *p* < 0.01, *** *p* < 0.001, and **** *p* < 0.0001 between indicated groups. ns denotes no statistical significance with *p* ≥ 0.05; **Table S5.** DNA Oligo Primer Sequences Used in qPCR.
