# Supplementary material for: Microbial Indoles: Key Regulators of Organ Growth and Metabolic Function
Source: Microorganisms. 2024 Apr 2;12(4):719. doi: 10.3390/microorganisms12040719 (PMC11052216; doi:10.3390/microorganisms12040719)
Supplement: Supplementary file 1 [file microorganisms-12-00719-s001.zip › microorganisms-2928939-supplementary.pdf]

SUPPLEMENTAL INFORMATION

FIGURE S1

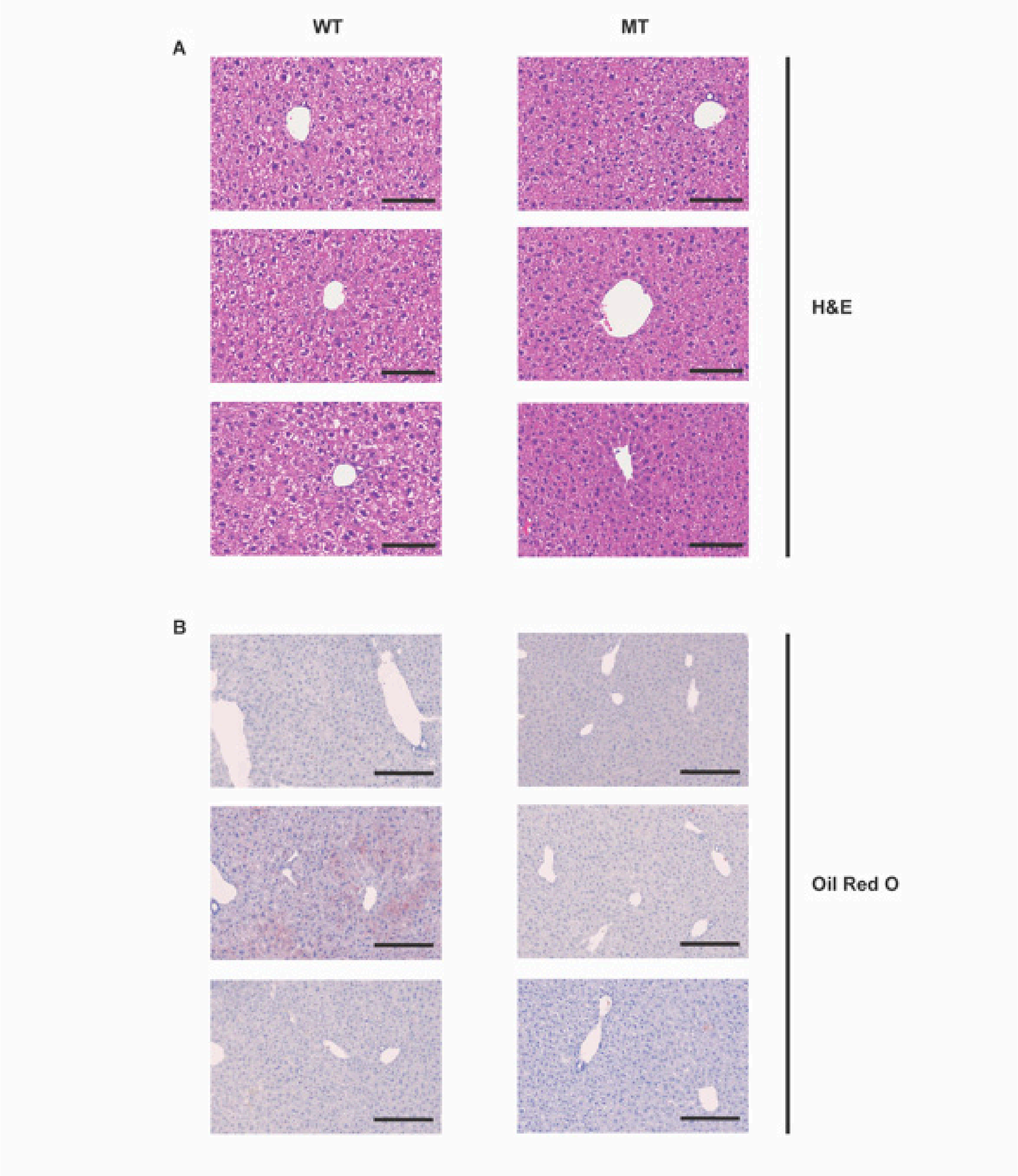

Figure S1. Representative images of liver histological analyses by (**A**) haematoxylin and eosin (H&E) and (**B**) oil red O staining. For **A**, scale bars = 100 μm; For **B**, scale bars = 200 μm

FIGURE S2

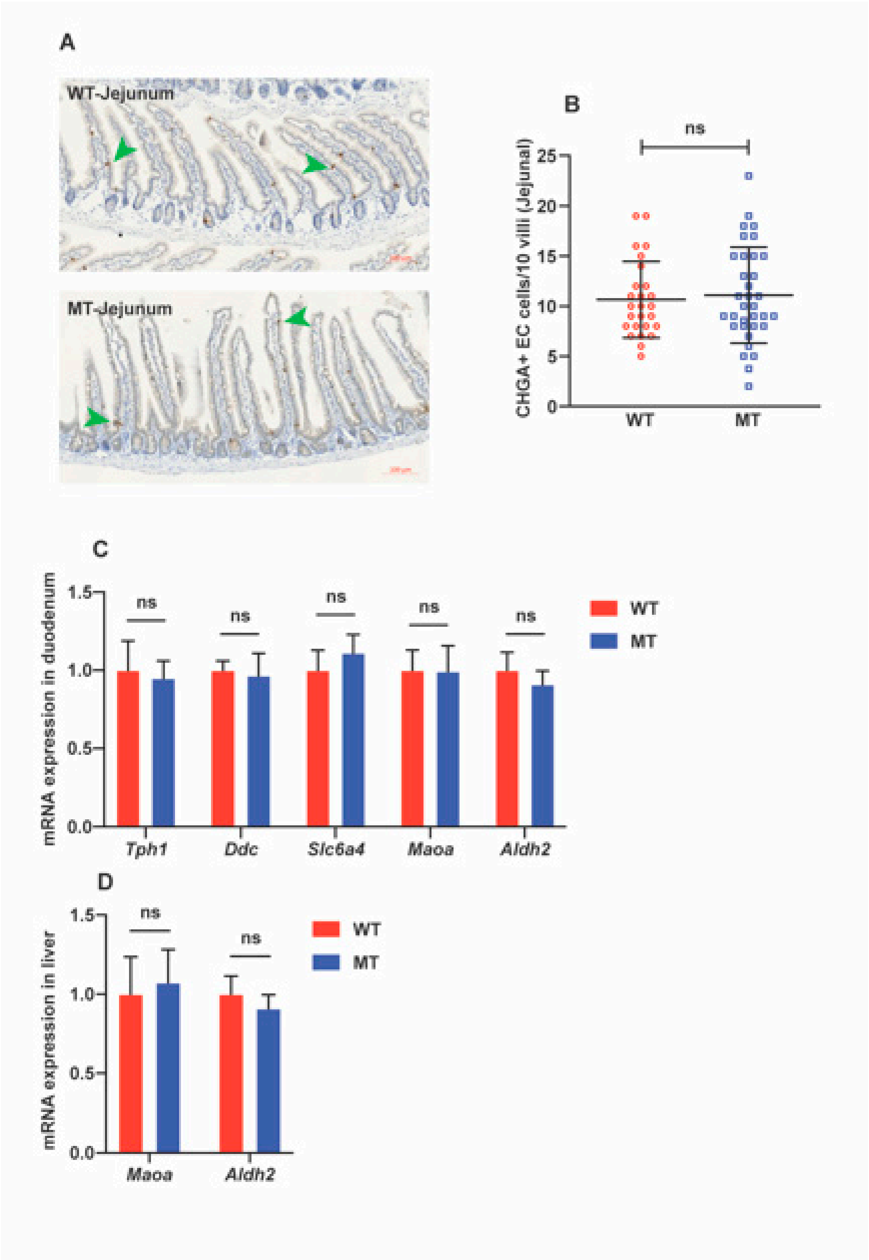

Figure S2. Jejunal serotonin-producing enterochromaffin cell numbers and serotonin metabolism in SI and liver in WT and MT mice. (**A**) Representative images of jejunal tissue sections stained with anti-chromogranin A (CHGA) antibody for enterochromaffin cells (in brown, indicated by green arrow heads), in jejuna of WT and MT mice, with haematoxylin-counterstained nuclei (in blue). Scale bar = 100 µm. (**B**) Quantification of $CHGA^+$ enterochromaffin cells (number of cells per 10 villi) in jejuna of WT and MT mice (n=6 per group). (**C**) mRNA expression of enzymes for serotonin synthesis *Tph1* (tryptophan hydroxylase 1) and *Ddc* (5-hydroxytryptophan decarboxylase), serotonin transporter *Slc6a4*, and enzymes for serotonin metabolism *Maoa* and *Aldh2*, in the duodena of WT and MT mice

(n=8 per group). (**D**) mRNA expression of enzymes for serotonin metabolism *Maoa* and *Aldh2* in the livers of WT and MT mice (n=8 per group). mRNA levels were normalized to the control gene *Hprt1*. Data graphs show means ± S.D. p values were calculated with the student's t test. ns denotes not significant with $p \geq 0.05$ between indicated groups.

FIGURE S3

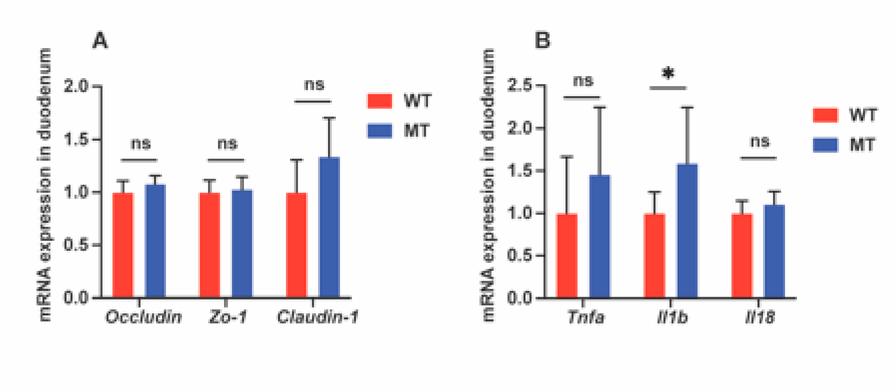

Figure S3. qPCR analyses on (**A**) intestinal tight junction proteins, *Occludin*, *Zo-1* (zonula occludens-1), and *Claudin-1* (n=8 per group); and (**B**) pro-inflammatory cytokines *Tnfa* (tumour necrosis factor alpha), *Il1b* (interleukin 1 beta), *Il18* (interleukin 18), in the duodena of WT and MT mice (n=7-8 per group). mRNA levels were normalized to the control gene *Hprt1*. Data graphs show means ± S.D. p values were calculated with the student's t test. Statistical significance is presented as $*p < 0.05$ between indicated groups. ns denotes not significant with $p \geq 0.05$.

FIGURE S4

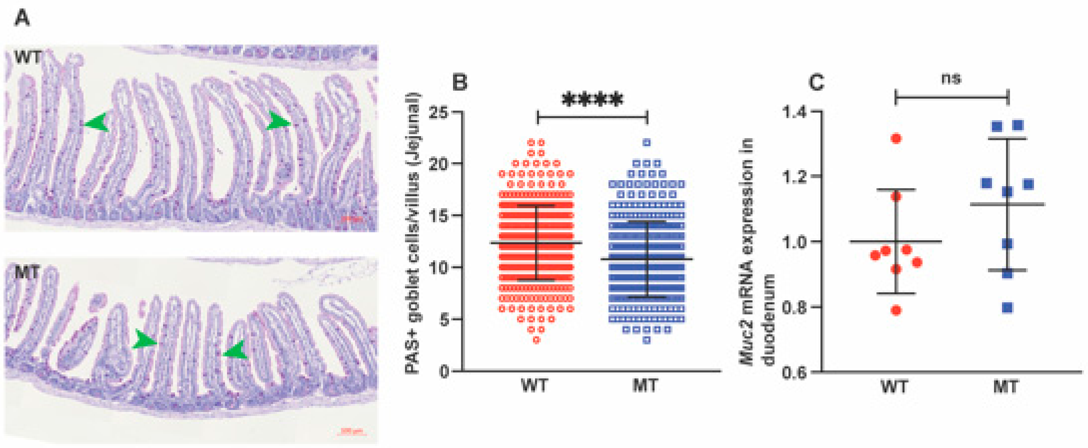

Figure S4. Indole-non-producing *E. coli*-colonized mice contained fewer mucus-producing goblet cells in the small intestines. (**A**) Representative images of jejunal tissue sections stained with periodic acid-Schiff (PAS) reagent for goblet cells (in deep purple, green arrow heads indicate representative cells), in jejuna of WT and MT mice, with haematoxylin-counterstained nuclei (in blue). Scale bar, 100 µm. (**B**) Quantification of goblet cells (number of PAS^+ cells per villus) in jejuna of WT and MT mice (n=6 per group). (**C**) mRNA expression of mucin 2 (*Muc2*) in the duodena of WT and MT mice (n=8 per group). mRNA levels were normalized to the control gene *Hprt1*. Data graphs show means ± S.D. *p* values were calculated with the student's *t* test. Statistical significance is presented as $^{****}p < 0.0001$ between indicated groups. ns denotes not significant with $p \geq 0.05$.

FIGURE S5

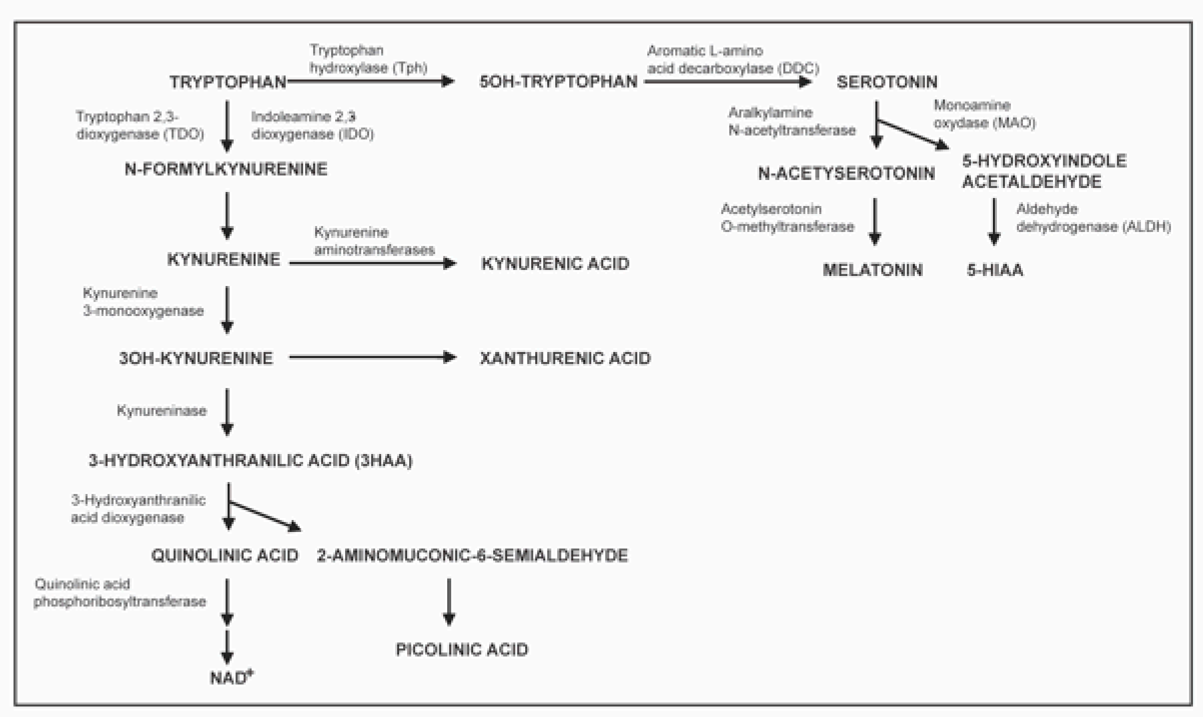

Figure S5. Illustration of tryptophan metabolisms in the mammalian host. Tryptophan can be metabolized in the host via the kynurenine pathway, through which kynurenine and NAD^+, along with other metabolites, are produced. Alternatively, tryptophan can also be metabolized in the intestines by the enterochromaffin cells, or in the brain, through the serotonin pathway, leading to the production of serotonin. Serotonin can be further metabolized to 5-hydroxyindoleacetic acid (5-HIAA) or be utilized as a substrate for production of melatonin, a hormone regulating the sleep-wake cycle. NAD^+, nicotinamide adenine dinucleotide (oxidized). Figure is adapted from Schwarcz & Stone, 2017[41]. Copyright © 2016 Elsevier Ltd. Adapted with permission.

Table S1. mRNA expression levels of selected genes in liver samples from WT and MT mice. n=7-8 per group. p values were calculated with the student's t test. Statistical significance is presented as $*p < 0.05$, $**p < 0.01$, $***p < 0.001$, and $****p < 0.0001$ between indicated groups. ns denotes no statistical significance with $p \geq 0.05$.

Functions	Genes	Gene products	Expression in MT mice compared with WT mice	Statistical significance	*p* values
Metabolism regulators	*Pgc1a*	Peroxisome proliferator-activated receptor gamma coactivator 1-alpha, a regulator of energy metabolism and mitochondrial biogenesis	-	ns	0.1093
	Sirt1	Sirtuin 1, a regulator of hepatic gluconeogenesis, fatty acid oxidation and cholesterol degradation	↑	**	0.0015
Glucose metabolism regulator	*Hnf4a*	Hepatocyte nuclear factor 4 alpha	↓	ns	0.1068
TCA cycle enzymes	*Pc*	Pyruvate carboxylase, converting pyruvate to oxaloacetate	↓	**	0.0046
	Cs	Citrate synthase, converting oxaloacetate to citrate	↓	****	<0.0001
	Aco2	Aconitase, converting citrate to *cis*-Aconitate	↓	*	0.0251
Lipogenesis enzymes	*Acly*	ATP citrate lyase, converting citrate to acetyl-CoA	↓	**	0.0096
	Acaca	Acetyl-CoA carboxylase (alpha), converting acetyl-CoA to malonyl-CoA (the rate-limiting step in fatty acid synthesis)	↓	*	0.0229
	Acacb	Acetyl-CoA carboxylase (beta)	↓	*	0.0146
	Fasn	Fatty acid synthase	↓	*	0.0167
	Hmgcs1	Hydroxymethylglutaryl-CoA synthase, second step enzyme of cholesterol synthesis pathway	↓	*	0.0429
	Hmgcr	Hydroxymethylglutaryl-CoA reductase, rate-controlling enzyme of cholesterol synthesis pathway	-	ns	0.2850
	Gyk	Glycerol kinase, an enzyme involved in lipogenesis	-	ns	0.5474
Lipolysis enzymes	*Acsl1*	long-chain fatty-acid-coenzyme A ligase	-	ns	0.3367
	Lipe	Hormone-sensitive lipase	-	ns	0.1510
	Pnpla2	Adipose triglyceride lipase	-	ns	0.1439
Lipid	*Ppara*	Peroxisome proliferator-	-	ns	0.2821

metabolism regulators		activated receptor alpha, a major regulator of lipid metabolism in the liver			
	Fiaf	Angiopoietin-like 4, a serum hormone regulating lipid metabolism	↑	**	0.0061
Serotonin metabolic enzymes	*Maoa*	Monoamine oxidase A, enzyme for first step of serotonin metabolism	-	ns	0.5229
	Aldh2	Aldehyde dehydrogenase, enzyme for second step of serotonin metabolism	↓	*	0.0240
Inflammation	*Il1b*	Interleukin 1B, a proinflammatory cytokine	-	ns	0.1731
	Il18	Interleukin 18, a proinflammatory cytokine	-	ns	0.3366
K+ channel	*Kcnj12*	Potassium Inwardly Rectifying Channel Subfamily J Member 12	↓	****	<0.0001

Table S2. mRNA expression levels of selected genes in epididymal white adipose tissue (WAT) samples from WT and MT mice. n=6-8 per group. *p* values were calculated with the student's *t* test. Statistical significance is presented as *$p < 0.05$, **$p < 0.01$, ***$p < 0.001$, and ****$p < 0.0001$ between indicated groups. ns denotes no statistical significance with $p \geq 0.05$.

Functions	Genes	Gene products	Expression in MT mice compared with WT mice	Statistical significance	*p* values
Metabolic regulators	*Pgc1a*	Peroxisome proliferator-activated receptor gamma coactivator 1-alpha	-	ns	0.8131
	Pparg	Peroxisome proliferator-activated receptor gamma, a regulator of fat storage and glucose metabolism	-	ns	0.4300
Glucose transporter	*Glut4*	Glucose transporter type 4	↑	*	0.0174
Glyceroneogenesis enzymes	*Pck1*	Phosphoenolpyruvate carboxykinase 1, cytosolic, an enzyme for glyceroneogenesis in adipose tissues	↑	*	0.0216
	Pck2	Phosphoenolpyruvate carboxykinase 2, mitochondrial, an enzyme for glyceroneogenesis in adipose tissues	↑	**	0.0027
Lipolysis enzymes	*Adrb2*	Adrenergic receptor beta-2, regulating lipolysis	↑	*	0.0323
	Adrb3	Adrenergic receptor beta-3, regulating lipolysis	↑	ns	0.1982
	Acsl1	long-chain fatty-acid-coenzyme A ligase	↑	**	0.0341
	Lpl	Lipoprotein lipase	-	ns	0.6162
	Lipe	Hormone-sensitive lipase	-	ns	0.9809
	Pnpla2	Adipose triglyceride lipase	-	ns	0.4106
	Pnpla3	Adiponutrin, a triacylglycerol lipase	-	ns	0.8219
	Fiaf	Angiopoietin-like 4, a serum hormone regulating lipid metabolism	-	ns	0.4988
Lipogenesis enzymes	*Scd1*	Stearoyl-CoA desaturase-1, catalysing the rate-limiting step in the formation of monounsaturated fatty acids	-	ns	0.9098
	Scd2	Stearoyl-CoA desaturase 2	↓	*	0.0294
	Fasn	Fatty acid synthase	↓	ns	0.9768
	Acaca	Acetyl-CoA carboxylase	-	ns	0.870

		(alpha)			2
	Acacb	Acetyl-CoA carboxylase (beta)	-	ns	0.532 2
Fatty acid transport	*Cd36*	Fatty acid translocase	-	ns	0.069 7
	Fatp1	Long-chain fatty acid transport protein 1	↑	**	0.002 0
	Fatp4	Long-chain fatty acid transport protein 4	↑	**	0.007 0
Heat production	*Ucp1*	Thermogenin	-	ns	0.084 8
	Ucp2	Mitochondrial uncoupling protein 2	-	ns	0.656 8
Inflammation	*Il1b*	Interleukin 1 beta, a proinflammatory cytokine	-	ns	0.157 4
	Il6	Interleukin 6, a pro-inflammatory cytokine and an anti-inflammatory myokine	↓	**	0.009 5
	Il18	Interleukin 18, a proinflammatory cytokine	-	ns	0.245 0
	Tnfa	Tumour necrosis factor, a cytokine for systemic inflammation	-	ns	0.487 6

Table S3. mRNA expression levels of selected genes in hindlimb muscles gastrocnemius (GS) samples from WT and MT mice. n=7-8 per group. *p* values were calculated with the student's *t* test. Statistical significance is presented as $*p < 0.05$, $**p < 0.01$, $***p < 0.001$, and $****p < 0.0001$ between indicated groups. ns denotes no statistical significance with $p \geq 0.05$.

Functions	Genes	Gene products	Expression in MT mice compared with WT mice	Statistical significance	*p* values
Metabolism regulators	*Pgc1a*	Peroxisome proliferator-activated receptor gamma coactivator 1-alpha, a regulator of energy metabolism and mitochondrial biogenesis	-	ns	0.9843
	Pparg	Peroxisome proliferator-activated receptor gamma, a regulator of fat storage and glucose metabolism	-	ns	0.3779
Glucose metabolism and mitochondrial functions	*Glut4*	Glucose transporter	↓	*	0.0285
	Sdh	succinate dehydrogenase, a TCA cycle and respiratory chain enzyme	↓	ns	0.1879
	Tfam	Mitochondrial transcription factor A	↓	*	0.0456
	Pdk4	Pyruvate dehydrogenase lipoamide kinase isozyme 4, mitochondrial, a regulator of glucose metabolism	-	ns	0.6495
Lipid metabolism	*Ppara*	Peroxisome proliferator-activated receptor alpha, a major regulator of lipid metabolism in the liver	-	ns	0.6454
	Fiaf	Angiopoietin-like 4, a serum hormone regulating lipid metabolism	-	ns	0.9469
	Srebp2	Sterol regulatory element-binding protein 2, a transcription factor regulating cholesterol homeostasis	↓	*	0.0138
Muscle components	*Myhc-1*	Myosin heavy chain 1	-	ns	0.5249
	Myhc-2a	Myosin heavy chain 2a	-	ns	0.5336
	Myhc-2b	Myosin heavy chain 2b	-	ns	0.4575
	Myhc-2d	Myosin heavy chain 2d	↓	*	0.0182
	Rapsyn	Receptor-associated protein of the synapse (neuromuscular junction-associated gene)	↓	ns	0.2569
Muscle atrophy	*Murf-1*	Muscle RING-finger protein-1, a muscle atrophy E3 ubiquitin ligase	↓	*	0.0215
	Atrogin-1	A muscle atrophy E3 ubiquitin ligase	↓	**	0.0055

Myogenesis	*Myod*	Myoblast determination protein 1, regulating muscle differentiation	↓	n.s.	0.1975
Inflammation	*Il6*	Interleukin 6, a pro-inflammatory cytokine and an anti-inflammatory myokine	-	ns	0.4321
K+ channel	*Kcnj12*	Potassium Inwardly Rectifying Channel Subfamily J Member 12	-	ns	0.3598

Table S4. mRNA expression levels of selected genes in small intestine (SI) samples from WT and MT mice. n=7-8 per group. *p* values were calculated with the student's *t* test. Statistical significance is presented as *$p < 0.05$, **$p < 0.01$, ***$p < 0.001$, and ****$p < 0.0001$ between indicated groups. ns denotes no statistical significance with $p \geq 0.05$.

Functions	Genes	Gene products	Expression in MT mice compared with WT mice	Statistical significance	*p* values
Gut barrier integrity	*Occludin*	Occludin, an epithelial cell tight junction protein	-	ns	0.1124
	Zo-1	Zonula occludens-1, an epithelial cell tight junction protein	-	ns	0.6254
	Claudin-1	Claudin-1, an epithelial cell tight junction protein	-	ns	0.0636
Inflammation	*Tnfa*	Tumour necrosis factor, a cytokine for systemic inflammation	-	ns	0.2304
	Il1b	Interleukin 1 beta, a proinflammatory cytokine	↑	*	0.0443
	Il18	Interleukin 18, a proinflammatory cytokine	-	ns	0.1732
	Cd68	Protein expressed by immune cells	-	ns	0.5714
Serotonin synthesis, transport, and metabolism	*Tph1*	Tryptophan hydroxylase 1, first-step enzyme in serotonin synthesis	-	ns	0.5121
	Ddc	Aromatic amino acid decarboxylase, second-step enzyme in serotonin synthesis	-	ns	0.5296
	Slc6a4	Serotonin transporter	-	ns	0.0962
	Maoa	Monoamine oxidase A, enzyme for first step of serotonin metabolism	-	ns	0.9137
	Aldh2	Aldehyde dehydrogenase, enzyme for second step of serotonin metabolism	-	ns	0.0934
Active intestinal stem cell markers	*Lgr5*	Leucine-rich G-protein-coupled receptor 5	↓	***	0.0007
	Olfm4	Olfactomedin 4	-	ns	0.2988
Goblet cell marker	*Muc2*	Mucin 2	-	ns	0.2306
Wnt signalling pathway (regulating stem cell self-renewal)	*Ctnnb1*	β-catenin, a protein involved in Wnt signalling, a pathway important for intestinal stem cell renewal	-	ns	0.3537
	Lrp6	Low-density lipoprotein receptor-related protein 6, a receptor in Wnt signalling	-	ns	0.7782
	Fzd7	Frizzled-7, a receptor in Wnt signalling	-	ns	0.2633

Notch signalling (modulating stem cell differentiation)	*Hes1*	A transcription factor interacts with Notch signalling, modulating secretory lineage differentiation in intestinal epithelium	-	ns	0.2309
Cell proliferation	*Mcc*	Colorectal mutant cancer protein, a negative regulator of cell cycle progression	↓	*	0.0171
	Pcna	Proliferating cell nuclear antigen	-	ns	0.0587
	cMyc	A transcription factor regulating many pro-proliferation genes	-	ns	0.6450
	Ccnd1	Cyclin D1, a protein required for progression through G1 phase of cell cycle	-	ns	0.4143
	Cdkn1a	Cyclin-dependent kinase (CDK) inhibitor 1, p21Cip1, inhibitor of cyclins/CDKs, including PCNA	-	ns	0.7205
Glucose metabolism	*Sirt1*	Sirtuin 1, a regulator of hepatic gluconeogenesis, fatty acid oxidation and cholesterol degradation	-	ns	0.7542
	Glut2	Glucose transporter 2	-	ns	0.5875
	Abcg5	Proteins for regulation of sterol absorption and excretion	-	ns	0.7158
	Abcg8		-	ns	0.1150
	Idh2	Isocitrate dehydrogenase [NADP], mitochondrial, a TCA cycle enzyme	↓	**	0.0046
Lipid metabolism	*Npc1l1*	A protein involved in cholesterol absorption	↓	*	0.0175
	Scarb1	Scavenger receptor class B type 1, a receptor involved in cholesterol transport	-	ns	0.5271
	Fatp4	Long-chain fatty acid transport protein 4	-	ns	0.9533
Neural signalling	*Npy*	Neuropeptide Y, modulating diverse neurological processes including food intake and fat storage	-	ns	0.2131
K+ channel	*Kcnj12*	Potassium Inwardly Rectifying Channel Subfamily J Member 12	↓	**	0.004

Supplemental Table 5. DNA Oligo Primer Sequences Used in qPCR.

Gene Name	Sequence (5'→3')	
Abcg5	Forward	AGGGCCTCACATCAACAGAG
	Reverse	GCTGACGCTGTAGGACACAT
Abcg8	Forward	CTGTGGAATGGGACTGTACTTC
	Reverse	GTTGGACTGACCACTGTAGGT
Acaca	Forward	ATGGGCGGAATGGTCTCTTTC
	Reverse	TGGGGACCTTGTCTTCATCAT
Acacb	Forward	CCTTTGGCAACAAGCAAGGTA
	Reverse	AGTCGTACACATAGGTGGTCC
Acly	Forward	CAGCCAAGGCAATTTCAGAGC
	Reverse	CTCGACGTTTGATTAACTGGTCT
Aco2	Forward	ATCGAGCGGGGAAAGACATAC
	Reverse	TGATGGTACAGCCACCTTAGG
Acsl1	Forward	TGCCAGAGCTGATTGACATTC
	Reverse	GGCATACCAGAAGGTGGTGAG
Actb	Forward	GGCTGTATTCCCCTCCATCG
	Reverse	CCAGTTGGTAACAATGCCATGT
Adrb2	Forward	GGGAACGACAGCGACTTCTT
	Reverse	GCCAGGACGATAACCGACAT
Adrb3	Forward	CCTTGGGCGAAACTGGTTG
	Reverse	GTTGGTGACAGCTAGGTAGCG
Ahr	Forward	GCCAGGACCAGTGTAGAGC
	Reverse	ATTCAGCGCCTGTAACAAGAA
Ahrr	Forward	GACCCTCCTCAGGTGGTGTT
	Reverse	AATGAAGCAGCGTGTCAAGAA
Aldh2	Forward	GACGCCGTCAGCAGGAAAA
	Reverse	CGCCAATCGGTACAACAGC
Alpi	Forward	AGGATCCATCTGTCCTTTGG
	Reverse	ACGTTGTATGTCTTGGACAG
Atrogin-1	Forward	ATGCACACTGGTGCAGAGAG
	Reverse	TGTAAGCACACAGGCAGGTC

Bmi1	Forward	CCAATGAAGACCGAGGAGAA
	Reverse	TTTCCGATCCAATCTGCTCT
Ccnd1	Forward	GCGTACCCTGACACCAATCTC
	Reverse	CTCCTCTTCGCACTTCTGCTC
Cd36	Forward	TTGTGGGGTTACAAAGATCCA
	Reverse	AGGATAAAACACACCAACTGTGG
Cd68	Forward	TGTCTGATCTTGCTAGGACCG
	Reverse	AGGAGAGTAACGGCCTTTTTG
Cdkn1a	Forward	CCTGGTGATGTCCGACCTG
	Reverse	CCATGAGCGCATCGCAATC
Chga	Forward	CACGGGAGACAGTGAGAGAG
	Reverse	TCCGACTGACCATCATCTTTCT
Claudin-1	Forward	TGGGTTTCATCCTGGCTTCT
	Reverse	TGTATCTGCCCGGTGCTTT
cMyc	Forward	CCCTATTTCATCTGCGACGAG
	Reverse	GAGAAGGACGTAGCGACCG
Cpt1a	Forward	CGCACATTACAAGGACATGG
	Reverse	GAAGAGCCGAGTCATGGAAG
Cs	Forward	GGACAATTTTCCAACCAATCTGC
	Reverse	TCGGTTCATTCCCTCTGCATA
Ctnnb1	Forward	ATGGAGCCGGACAGAAAAGC
	Reverse	CTTGCCACTCAGGGAAGGA
Cyp1a1	Forward	CAATGAGTTTGGGGAGGTTACTG
	Reverse	CCCTTCTCAAATGTCCTGTAGTG
Cyp1a2	Forward	AGTACATCTCCTTAGCCCCAG
	Reverse	GGTCCGGGTGGATTCTTCAG
Cyp1b1	Forward	CACCAGCCTTAGTGCAGACAG
	Reverse	GAGGACCACGGTTTCCGTTG
Ddc	Forward	AGCTGGTTGCTTACACATCTG
	Reverse	CGAAGGGACTGCTTTTAGCTT
Fasn	Forward	TGGTGAATTGTCTCCGAAAAGA
	Reverse	CACGTTCATCACGAGGTCATG

Fatp1	Forward	CGCTTTCTGCGTATCGTCTG
	Reverse	GATGCACGGGATCGTGTCT
Fatp4	Forward	CTGTTTGCTTCAATGGTACAGC
	Reverse	CGGAAGGTCCAGTGAGTGTC
Fiaf	Forward	CAATGCCAAATTGCTCCAATT
	Reverse	TGGCCGTGGGCTCAGT
Fzd7	Forward	AGACCCACCTTTCACTGCG
	Reverse	AAGTACATGAGGCCGTTAGCA
Glut2	Forward	TCAGAAGACAAGATCACCGGA
	Reverse	GCTGGTGTGACTGTAAGTGGG
Glut4	Forward	GTGACTGGAACACTGGTCCTA
	Reverse	CCAGCCACGTTGCATTGTAG
Gyk	Forward	GTCAGCAACCAGAGGGAAACC
	Reverse	CCACGGCATTATAGAGAGGCT
Hes1	Forward	GATAGCTCCCGGCATTCCAAG
	Reverse	GCGCGGTATTTCCCCAACA
Hmgcs1	Forward	AAATGCCAGACCTACAGGTGG
	Reverse	ATGCTGCATGTGTGTCCCA
Hmgcr	Forward	AGCTTGCCCGAATTGTATGTG
	Reverse	TCTGTTGTGAACCATGTGACTTC
Hnf4a	Forward	GGTTTAGCCGACAATGTGTGG
	Reverse	TCCCGCTCATTTTGGACAGC
Idh2	Forward	GGAGAAGCCGGTAGTGGAGAT
	Reverse	GGTCTGGTCACGGTTTGGAA
Il1b	Forward	GAAATGCCACCTTTTGACAGTG
	Reverse	TGGATGCTCTCATCAGGACAG
Il6	Forward	CTGCAAGAGACTTCCATCCAG
	Reverse	AGTGGTATAGACAGGTCTGTTGG
Il18	Forward	GACTCTTGCGTCAACTTCAAGG
	Reverse	CAGGCTGTCTTTTGTCAACGA
Ki67	Forward	ATCATTGACCGCTCCTTTAGGT
	Reverse	GCTCGCCTTGATGGTTCCT

Kcnj12		PrimePCR SYBR Green Assay – Bio-Rad. Catalog # 10029101
Lgr4	Forward	GCAGTCTCTACGCTTAGATGC
	Reverse	CACTTCCGTCAAGATGTTGTCA
Lgr5	Forward	ACATTCCCAAGGGAGCGTTC
	Reverse	ATGTGGTTGGCATCTAGGCG
Lipe	Forward	CCAGCCTGAGGGCTTACTG
	Reverse	CTCCATTGACTGTGACATCTCG
Lpl	Forward	TTGCCCTAAGGACCCCTGAA
	Reverse	TTGAAGTGGCAGTTAGACACAG
Lrp6	Forward	TTGTTGCTTTATGCAAACAGACG
	Reverse	GTTCGTTTAATGGCTTCTTCGC
Lyz	Forward	GTCACACTTCCTCGCTTTCC
	Reverse	TGGCTTTGCTGACTGACAAG
Maoa	Forward	GCCCAGTATCACAGGCCAC
	Reverse	CGGGCTTCCAGAACCAAGA
Mcc	Forward	CGACGTTGACAGGCACATAGA
	Reverse	GCCAGGTTGGGATACAGATCG
Muc2	Forward	CCGACTTCAACCCAAGTGAT
	Reverse	GAGCAAGGGACTCTGGTCTG
Murf-1	Forward	AGAAGTCGGGGGTCAGGGGACG
	Reverse	GGTCCATGATCACTTCATGGCGGCACGAGG
Myhc-1	Forward	CCAAGGGCCTGAATGAGGAG
	Reverse	GCAAAGGCTCCAGGTCTGAG
Myhc-2a	Forward	AAGCGAAGAGTAAGGCTGTC
	Reverse	GTGATTGCTTGCAAAGGAAC
Myhc-2b	Forward	ACAAGCTGCGGGTGAAGAGC
	Reverse	CAGGACAGTGACAAAGAACG
Myhc-2d	Forward	CCAAGTGCAGGAAAGTGACC
	Reverse	AGGAAGAGACTGACGAGCTC
Myod	Forward	TACAGTGGCGACTCAGATGC
	Reverse	GAGATGCGCTCCACTATGCT
Npc1l1	Forward	CGCCCTTCTTTCTACATGGGT

	Reverse	GAATCTGCGCTTACGAGGGAG
Npy	Forward	TACTCCGCTCTGCGACACTA
	Reverse	TCTTCAAGCCTTGTTCTGGG
Occludin	Forward	GCAGCCTTCTGCTTCATCG
	Reverse	CGTCGGGTTCACTCCCATTA
Olfm4	Forward	CAGCCACTTTCCAATTTCACTG
	Reverse	GCTGGACATACTCCTTCACCTTA
Pc	Forward	CAGTGGCTGTCTACTCGGAG
	Reverse	CCGCATCTACACCATTTTCCT
Pck1	Forward	TGACAGACTCGCCCTATGTG
	Reverse	CCCAGTTGTTGACCAAAGGC
Pck2	Forward	ATGGCTGCTATGTACCTCCC
	Reverse	GCGCCACAAAGTCTCGAAC
Pcna	Forward	ACCTCACCAGCATGTCCAA
	Reverse	TCTTGATTTGGTGCTTCGAATA
Pdk4	Forward	CCGCTTAGTGAACACTCCTTC
	Reverse	TGACCAGCGTGTCTACAAACT
Pgc1a	Forward	AAGGGTTCCCCATTTGAGA
	Reverse	AGGGTTATCTTGGTTGGCTTT
Pnpla2	Forward	TCCGTGGCTGTCTACTAAAGA
	Reverse	TGGGATATGATGACGTTCTCTCC
Pnpla3	Forward	AGGTCATTTCTGGCAAGGTTC
	Reverse	TCCACGACTTCGTCTTTGGAA
Ppara	Forward	AACATCGAGTGTCGAATATGTGG
	Reverse	CCGAATAGTTCGCCGAAAGAA
Pparg	Forward	TCGCTGATGCACTGCCTATG
	Reverse	GAGAGGTCCACAGAGCTGATT
Rapsyn	Forward	GGCAGGACCAGACAAAGCAA
	Reverse	CGAGTGAGCTGTTACCAAGCA
Scarb1	Forward	TGTACTGCCTAACATCTTGGTCC
	Reverse	ACTGTGCGGTTCATAAAAGCA
Scd1	Forward	TTCTTGCGATACACTCTGGTGC

	Reverse	CGGGATTGAATGTTCTTGTCGT
Scd2	Forward	GATCTCTGGCGCTTACTCAGC
	Reverse	CTCCCCAGTGGTGAGAACTC
Sdh	Forward	GAAAGGCGGGCAGGCTCATC
	Reverse	CACCACGGCACTCCCCATTTT
Sirt1	Forward	GCTGACGACTTCGACGACG
	Reverse	TCGGTCAACAGGAGGTTGTCT
Slc2a4	Forward	GGACCGGATTCCATCCCAC
	Reverse	TCCCAACCATTGAGAAATGATGC
Slc6a4	Forward	GTCATTGGCTATGCCGTGGA
	Reverse	CACCCATTTCGGTGGTACTG
Slc25a1	Forward	GAGGCATCGAAATCTGCATCA
	Reverse	GGATGGAGCCGTAGAGCAA
Srebp2	Forward	CCAAAGAAGGAGAGAGGCGG
	Reverse	CGCCAGACTTGTGCATCTTG
Tfam	Forward	GCTCTACACGCCCCTGGTTTCTGG
	Reverse	TCGCTGTAGTGCCTGCTGCTCCTG
Tnfa	Forward	GTCCCCAAAGGGATGAGAAGT
	Reverse	TTTGCTACGACGTGGGCTAC
Tph1	Forward	AACAAAGACCATTCCTCCGAAAG
	Reverse	TGTAACAGGCTCACATGATTCTC
Ucp1	Forward	AGGCTTCCAGTACCATTAGGT
	Reverse	CTGAGTGAGGCAAAGCTGATTT
Ucp2	Forward	ATGGTTGGTTTCAAGGCCACA
	Reverse	CGGTATCCAGAGGGAAAGTGAT
Vil1	Forward	GGCAACGAGAGCGAGACTTT
	Reverse	GGAGTTTGTTTCTACGTGCTTCA
Zo-1	Forward	TCACGATCTCCTGACCAACG
	Reverse	GGCTGACGGGTAAATCCACA
